# Genetic transformation of *Fusarium avenaceum* by *Agrobacterium tumefaciens* mediated transformation and the development of a USER-Brick vector construction system

**DOI:** 10.1186/1471-2199-15-15

**Published:** 2014-07-22

**Authors:** Lisette Quaade Sørensen, Erik Lysøe, Jesper Erup Larsen, Paiman Khorsand-Jamal, Kristian Fog Nielsen, Rasmus John Normand Frandsen

**Affiliations:** 1 Eukaryotic Molecular Cell Biology Group, Department of Systems Biology, The Technical University of Denmark, Søltofts Plads building 223, DK-2800 Kgs., Lyngby, Denmark; 2 Metabolic Signaling and Regulation group, Department of Systems Biology, The Technical University of Denmark, Søltofts Plads building 221, DK-2800 Kgs., Lyngby, Denmark; 3 Bioforsk–Norwegian Institute of Agricultural and Environmental Research, Høgskoleveien 7, Ås 1430, Norway

**Keywords:** Single step cloning, ATMT, *Agrobacterium tumefaciens* mediated transformation, *Fusarium avenaceum*, USER-Brick, Genome modification, Transformation, Fusaristatin, *FaPKS6*, Mycotoxin, LC-MS, MS-MS, Polyketide, Nonribosomal peptide

## Abstract

**Background:**

The plant pathogenic and saprophytic fungus *Fusarium avenaceum* causes considerable in-field and post-field losses worldwide due to its infections of a wide range of different crops. Despite its significant impact on the profitability of agriculture production and a desire to characterize the infection process at the molecular biological level, no genetic transformation protocol has yet been established for *F. avenaceum*. In the current study, it is shown that *F. avenaceum* can be efficiently transformed by *Agrobacterium tumefaciens* mediated transformation. In addition, an efficient and versatile single step vector construction strategy relying on Uracil Specific Excision Reagent (USER) Fusion cloning, is developed.

**Results:**

The new vector construction system, termed USER-Brick, is based on a limited number of PCR amplified vector fragments (core USER-Bricks) which are combined with PCR generated fragments from the gene of interest. The system was found to have an assembly efficiency of 97% with up to six DNA fragments, based on the construction of 55 vectors targeting different polyketide synthase (PKS) and PKS associated transcription factor encoding genes in *F. avenaceum*. Subsequently, the Δ*FaPKS3* vector was used for optimizing *A. tumefaciens* mediated transformation (ATMT) of *F. avenaceum* with respect to six variables. Acetosyringone concentration, co-culturing time, co-culturing temperature and fungal inoculum were found to significantly impact the transformation frequency. Following optimization, an average of 140 transformants per 10^6^ macroconidia was obtained in experiments aimed at introducing targeted genome modifications. Targeted deletion of *FaPKS6* (FA08709.2) in *F. avenaceum* showed that this gene is essential for biosynthesis of the polyketide/nonribosomal compound fusaristatin A.

**Conclusion:**

The new USER-Brick system is highly versatile by allowing for the reuse of a common set of building blocks to accommodate seven different types of genome modifications. New USER-Bricks with additional functionality can easily be added to the system by future users. The optimized protocol for ATMT of *F. avenaceum* represents the first reported targeted genome modification by double homologous recombination of this plant pathogen and will allow for future characterization of this fungus. Functional linkage of *FaPKS6* to the production of the mycotoxin fusaristatin A serves as a first testimony to this.

## Background

The plant pathogenic fungus *Fusarium avenaceum* displays a wide host range and causes diseases such as root rot and ear blight of cereals [1]. The financial losses are mainly due to crown rot and head blight of wheat and the accompanying contamination of the harvested grains with mycotoxins [2]. In addition, to the direct in field effects on harvest yields, this species is also responsible for post-harvest rot of many crops, such as swede turnip [3], apple [4], broccoli [5] and potato tubers [6]. In temperate regions of the world, such as Scandinavia, Russia and Canada, *F. avenaceum* has in several reports been found to be the dominant species in connection with head blight. However, in recent years an increasing prevalence in warmer regions, throughout the world, has also been reported [2,7]. During infections and in post-harvest situations *F. avenaceum* has been reported to produce various bioactive secondary metabolites, including antibiotic Y, chlamydosporol, aurofusarin, enniatins, and the mycotoxins fusarin C, 2-amino-14,16-dimethyloctadecan-3-ol, and moniliformin [2,8].

In spite of its significant impact on agricultural production only a few studies have aimed at elucidating the molecular genetic basis of this species’ broad host range, large geographical distribution and potential for biosynthesis of secondary metabolites [9]. One of the major hurdles for such studies in this species, and many others is the lack of reliable genetic transformation protocols and a basic toolbox for performing targeted genome modifications. The *Agrobacterium tumefaciens* mediated transformation (ATMT) technique has proven capable of transforming a wide range of different fungal species [10].

The current study concerns the establishment of an efficient transformation protocol for *F. avenaceum*, via ATMT, and the development of a new strategy for single step construction of binary vectors compatible with ATMT of filamentous fungi. The new USER-Brick vector system is compatible with two different popular vector backbone series, pAg1 and ppPK2/pPZP-201BK, and allows for high throughput vector construction. The system provides a versatile toolbox for the construction of plasmids that can be used to address typically posed biological questions, such as 1) what effect will deletion or 2) overexpression of a given gene have on the phenotype, 3) when, and where in the mycelium is the gene in question expressed, and 4) the subcellular localization of a given protein. The system was tested by performing targeted modification (deletion and overexpression) of fourteen different loci in the *F. avenaceum* genome, and by overexpression of 30 PKS associated transcription factor encoding genes from random loci in the *F. avenaceum* genome.

## Methods

### Organisms

*E. coli* DH5α was used in connection with USER cloning experiments. Chemically competent *E. coli* cells were produced as described in [11]. *Agrobacterium tumefaciens* LBA4404 was used for ATMT experiments. Transformation and cultivation of *E. coli* and *A. tumefaciens* was performed as described in [12]. *F. avenaceum* IBT41708 and *Fusarium graminearum* PH-1 were obtained from the fungal culture collection at The Technical University of Denmark. Macroconidia for both fungal species were produced using sporulation medium as described in [13]. For the selective steps of ATMT the fungi were cultured at 26°C in darkness on Defined Fusarium Medium (DFM): 12.5 g/L glucose, 1.32 g/L L-asparagine, 0.517 g/L MgSO_4_, 1.524 g/L KH_2_PO_4_, 0.746 g/L KCl, 0.04 mg/L Na_2_B_4_O_7_, 0.4 mg/L CuSO_4_, 1.2 mg/L FeSO_4_, 0.7 mg/L MnSO_4_, 0.8 mg/L NaMoO_2_, 10 mg/L ZnSO_4_.

### Amplification of USER-Bricks

**Figure 1:**
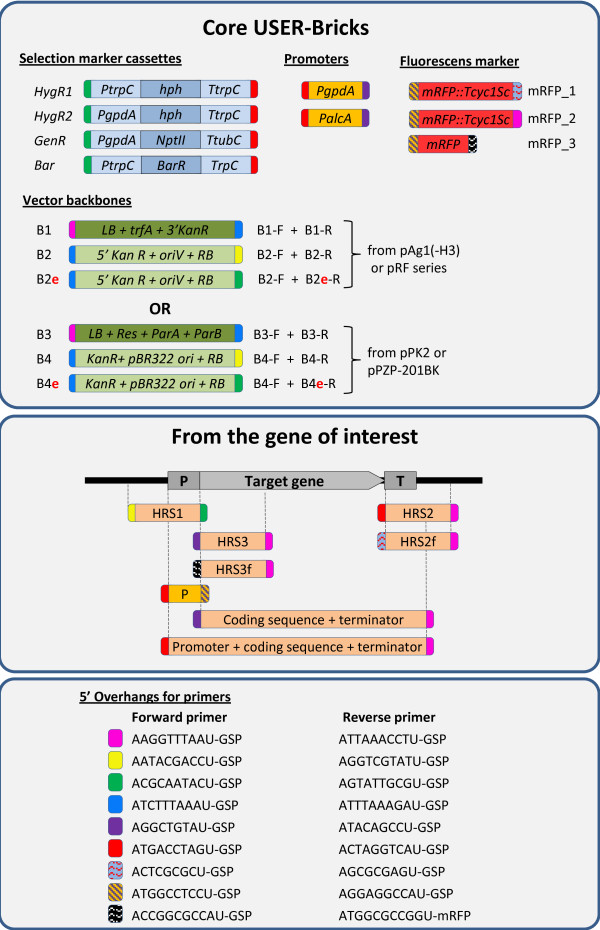
**The different DNA fragments (=bricks) in the USER-Brick vector system.** The ends of the Bricks are colour coded based on which overhangs that are compatible for fusion. ***Top panel***: The core USER-Brick includes backbones, selection markers, promoters and fluorescent marker fragments. ***Centre panel***: The placement of the different types of PCR amplicons in relation to the gene of interest. ***Bottom panel***: Sequences of the 5′ overhang found on the primers for amplifying the different USER-Bricks in the two panels above.

**Figure 2:**
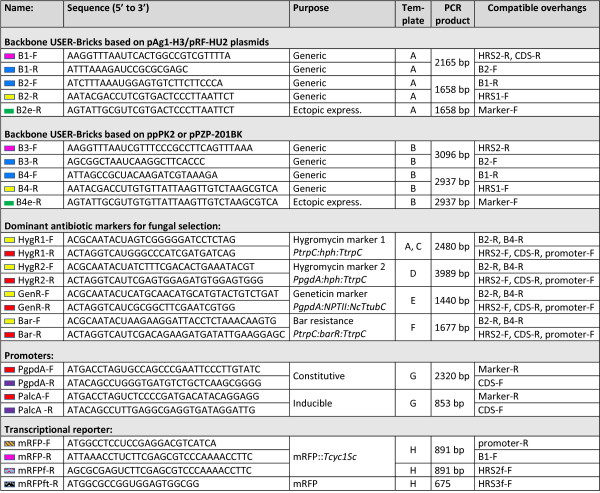
**Primer sequences for amplifying the different USER-Bricks, specifying template, product size and compatible USER-Brick fragments. ***PCR templates*: A = pAg1-H3, pRF-HU2 [15], B = pPK2 or pPZP-201BK [16], C = pANT-hyg(R) [17] or pCSN43 (Fungal Genetics Stock Center), D = pAN7-1 [18], E = pSM334 [19], F = pBARKS1 [20], G = *A. nidulans genomic DNA* or pRF-HUE, pRF-HU2E [15], H = pWJ1350 [21] or plasmids derived from the original *Discosoma* sp. study [22]. In primer sequences: U = 2-deoxyuridine.

**Figure 3:**
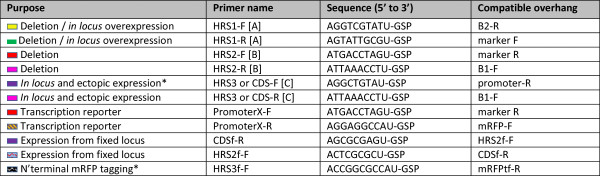
**Primer overhangs that should be added to the 5’ end of the gene specific primers to allow for construction of the specified types of vector constructs.** A = natural promoter regions of the target gene; B = the terminator region of the gene and C: for *in locus* overexpression experiments the first 1500 bp of the gene so that the AU in the forward primer (HRS3, CDS-F and HRS3f) is part of the start codon, and for ectopic expression experiments so that the entire coding sequence and terminator are amplified.

The current core USER-Bricks includes vector backbones, selection markers, promoters and fluorescent markers as shown in Figure 1 top panel. PCR reactions were performed using X7 [14] or Pfu Turbo Cx Hotstart DNA polymerase (Agilent Technologies) in 50 μl reactions with Phusion HF buffer (New England Biolabs). Core USER-Bricks were amplified from purified plasmid DNA, 1 ng/50 μl reaction, using 2-deoxyuridine containing primers from Integrated DNA Technologies (Leuven, Belgium) as specified in Figure 2. Genomic inserts (Figure 1, centre panel) unique for the individual target were amplified from purified *F. avenaceum* genomic DNA, 10 ng/50 μl reactions, using the gene specific primers listed in Additional file 1: Tables S1 and S2, with the relevant overhangs specified in Figure 3. For targeted integration 1500 bp long homologous recombination sequences were used, and in the case of expression from random loci the coding sequence of the gene plus 500 bp downstream were amplified. A G-Storm GS2 thermal cycler (G-Storm, Somerton, UK) was used for PCR, with the following program: 98°C for 10 min, 35 cycles (98°C for 20 sec, 60°C for 20 sec, 72°C for 2 min), 72°C for 5 min. The PCR amplicons were gel purified using the illustra GFX PCR DNA and Gel Band Purification Kit (GE Healthcare), to eliminate the plasmid and genomic DNA that had served as the templates during the PCR reactions.

### Construction of plasmids from USER-Bricks

The USER-Bricks and ‘gene specific fragments’ needed for the different types of experiments are described in Figure 1. For USER cloning reactions 1 μl of the needed purified USER-Bricks and 2 μl of the required gene specific inserts were mixed with 1.2 units of ‘USER enzyme mix’ (New England Biolabs) and 10xTaq DNA polymerase buffer (Sigma-Aldrich) to a final concentration of 1x, in a total volume of 12 μl. The reactions were incubated for 25 min at 37°C and then for 25 min at 25°C using a thermal cycler. The entire reaction volume was used for transformation of 50 μl chemically competent *E. coli* DH5α cells, as described in [12]. Ten of the resulting transformants were analysed by colony-PCR, using the gene specific primers, and restriction enzyme digestion to verify correct assembly. In cases where the plasmids did not yield the expected results, the presence of the core USER-Bricks was tested by PCR.

**Figure 4:**
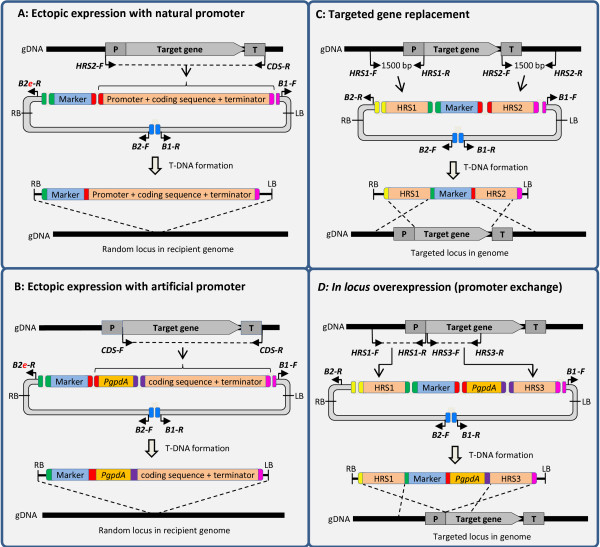
**Design of vectors for random heterologous expression with the gene’s natural promoter (A), with an alternative promoter (B), for targeted gene replacement (C) and *in locus *overexpression (D). A)** Expression of the gene of interest from a random locus in the genome, driven by the gene’s natural promoter. Note the use of the B2e USER-Brick to allow for direct fusion of the selection marker cassette with the B2 vector backbone. **B)** Overexpression of the gene of interest from a random genomic locus, with the expression driven by a heterologous promoter, in this case the *gpdA* promoter from *Aspergillus nidulans*. Note the use of the B2e USER-Brick to allow for direct fusion of the selection marker cassette with the B2 vector backbone. **C)** Replacement of the gene of interest. Note that the HRS1 fragment can also be reused for in locus overexpression experiments. **D)***In locus* overexpression of the gene of interest by targeted integration of a strong constitutive promoter. Note that the HRS1 fragment can be reused for deletion experiments. Primers are represented by solid black arrows. Aberrations: gDNA = genomic DNA; P = promoter; CDS = coding sequence; T = terminator; RB & LB = right & left borders defining the T-DNA region; T-DNA = transfer DNA.

**Figure 5:**
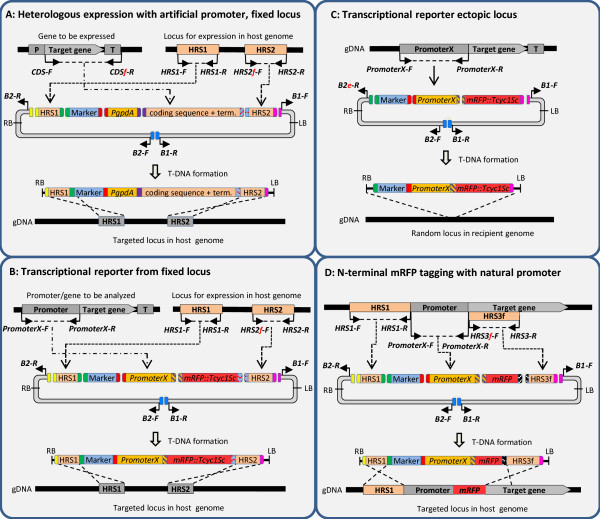
**Design of vectors for heterologous expression (A), transcriptional report constructs (B and C) and N’terminal mRFP tagging (D). A)** Heterologous expression of a gene of interest from a predefined locus in the genome. Note the unique overhangs on the HRS2f (targeted locus) and CDSf-R (gene to be expressed) fragments that allow for fusion of the two. **B)** Targeted integration of transcriptional reporter construct to monitor the expression of the gene of interest. **C)** Transcriptional reporter construct for random integration. **D)** N’terminal mRFP tagging of gene of interest. The promoter element in the setup can either be the gene’s natural promoter, which should be as short as possible to limit the change of recombination, or one of the heterologous promoters (*PgpdA* or *PalcA*). Primers are represented by solid black arrows. Aberrations: gDNA = genomic DNA; P = promoter; CDS = coding sequence; T = terminator; RB & LB = right & left borders defining the T-DNA region; T-DNA = transfer DNA.

The seven different types of vector constructs that the USER–Brick system currently allows for are shown in Figures 4 and 5, including information on which fragments should be combined in the individual case. Additional file 1: Table S1 summarizes the USER Bricks needed for the different constructions. These can either be constructed using the pAg1-H3/pRF-HU2 (B1 + B2) or the ppPK2/pPZP-201BK (B3 + B4) vector backbones, but note that the two vector backbone systems are not mixable. For the construction of ‘targeted deletion’ vectors, exemplified with the B1 + B2 backbone, the following was combined: B1 + B2 + HygR1 + upstream homologous recombination sequence (HRS) + downstream HRS, where the two HRS surround the target (Figure 4C and Additional file 1: Table S2). For ‘*in locus* overexpression’ constructions: B1 + B2 + HygR1 + PgpdA + upstream HRS + downstream HRS, where the downstream HRS includes the start of the gene to be overexpressed (Figure 4D and Additional file 1: Table S2). For overexpression of genes from a random locus in the recipient fungus genome, B1 + B2e + HygR1 + PgpdA + CDS + promoter element were combined (Figure 4B and Additional file 1: Table S3). Figure 4 also describes the construction of vectors for random ectopic expression using the gene’s natural promoter (Figure 4A). Figure 5, shows the strategies for expression from a predefined (fixed) locus in the genome (Figure 5A), transcriptional reporters from random (Figure 5C) and fixed loci (Figure 5B) and N’terminal mRFP tagging (Figure 5D). For experiments comparing the effect of the vector backbone a pPK2::Δ*FaPGL1/PKS3* plasmid was constructed (USER-Bricks: B3 + B4 + HygR1 + FaPKS3-U1/U2 + FaPKS3-U3/U4).

### Optimizing ATMT of *F. avenaceum*

The effects of hygromycin B (Invivogen), geneticin/G418 (Invivogen) and DL-phosphinothricin/Basta (Life Science) on germination of *F. avenaceum* was tested with the purpose of identifying the minimal concentration that allowed efficient inhibition during ATMT experiments. Five different concentrations of the three antibiotics were tested, hygromycin B: 75, 100, 150, 200, 250 μg/ml; geneticin: 150, 200, 250, 300, 350 μg/ml and for DL-phosphinothricin (Basta™) 200, 400, 600, 800, 1000 μg/ml. Macroconidia were initially plated onto black filter papers on IMAS medium, incubated for 48 hours and then transferred to DFM medium with the specified selection regimes. Following 10 days of incubation at 26°C in darkness, the amount of mycelium on and beneath the filters was evaluated.

ATMT was optimized using the pAg1-H3/pRF-HU2 vector series in the *A. tumefaciens* LBA4404 strain background with respect to six parameters. In these experiments, unless otherwise specified, the *A. tumefaciens* strain was pre-induced with 200 μM acetosyringone until OD_600_ 0.7, mixed 1:1 with 5*10^5^ macroconidia per agar plate, co-cultured for 2 days at 28°C. The following parameters were the subject of optimization: pre-induction of *A. tumefaciens* (+/− acetosyringone), acetosyringone concentration during co-culturing (0, 200 and 500 μM), co-cultivation time (24, 48, 72 and 96 hours), co-cultivation temperature (24°C, 26°C and 28°C), *F. avenaceum* inoculum (8×10^4^, 2×10^5^, 5×10^5^ and 1×10^6^ macroconidia/agar plate). The optimization was performed with three biological replicates, each with ten technical replicates (plates). The average number of obtained transformants per 10^6^ used spores was compared using a two tailed Student’s *T*-Test assuming unequal variances, performed in Microsoft Excel 2010. The pRF-HU2::Δ*FaPGL1/PKS3* plasmid, targeting the *PKS3*/*PGL1* locus, was used for all experiments, if not otherwise stated. For testing geneticin and DL-phosphinothricin based selection markers pRF-GU2::Δ*FaPGL1/PKS3* and pRF-BU2::Δ*FaPGL1/PKS3* plasmids were constructed.

Following optimization the system was used for performing targeted modification of thirteen different loci spread across the *F. avenaceum* genome and for random integration of 30 different overexpression constructs from random loci. In these experiments *A. tumefaciens* was grown to OD_600_ of 0.7 in LB media supplemented with kanamycin, co-cultivation was performed for 72 hours at 26°C, using 2×10^5^ macroconidia per plate, and selection with 150 μg/ml hygromycin B for six days.

### Targeted gene replacement in *F. graminearum*

The gene targeting efficiency in *F. graminearum* was assessed by targeted integration into the *PKS3/PGL1* (FGSG_17168) locus using a pRF-HU2::Δ*FgPGL1/PKS3* plasmid containing the hygromycin resistance marker (HygR1) flanked by two 1500 bp homologous recombination sequences amplified from the *F. graminearum* genome. The ATMT was performed as described in [23].

### PCR based genotyping of transformants from targeted genome modification experiments

In the experiments aimed at comparing gene targeting efficiency at the *PGL1/PKS3* locus in *F. avenaceum* and *F. graminearum*, 100 *F. avenaceum* and 104 *F. graminearum* transformants were randomly selected for PCR genotyping using four different primer pairs (described below). The targeting efficiency for the thirteen other targeted loci (deletions and *in locus* overexpression) in the *F. avenaceum* genome was determined by PCR genotyping of ten randomly selected transformants.

Genomic DNA for the study was obtained by transferring a small scrape of mycelium to a 1.5 ml Eppendorf tube with 100 ml 10:1 TE buffer, which was cooked for 10 minutes in a 750 Watt microwave oven at full effect. The supernatant was then diluted 100 times with MilliQ water, and 1 μl was used as a template in 15 μl PCR reactions with the different test primers (Additional file 1: Table S4 and S5). The different transformants were initially tested using a primer pair targeting the introduced dominant selection marker, with the purpose of verifying that the diluted DNA was of PCR grade. For deletion constructs; a primer pair (T1/T2) amplifying an internal fragment of the gene that was targeted for replacement was then used, with the purpose of testing for gene replacement. For deletion and *in locus* constructs, two different primer pairs, each bridging one of the used targeting sequences (homologous recombination), were used to test whether homologous recombination had occurred. For deletions the RF-1/T3 and RF-2/T4 primers were used and for *in locus* the RF-3/T1 and RF-2/T2 primers were used (Additional file 1: Table S4). For ectopic expression constructs; two primer pairs (RF-3/T1 and T2/’PKS12-A4/A4-T1’) were used to test for the presence of the start and end of the expression cassette (Additional file 1: Table S5). The PCR results were analysed by agarose gel electrophoresis (RunOne system, Embi Tec) or automated capillary electrophoresis (LabChip GX, PerkinElmer) (Table 1).

**Table 1:** Standard test primers for the analysis of the obtained transformants

**Primer name**	**Sequence (5′ to 3′)**	**Amplicon**	**Target**
**Primers for testing of the selection markers**			
HygR-T-F	AGCTGCGCCGATGGTTTCTACAA	588 bp	Test for marker gene
HygR-T-R	GCGCGTCTGCTGCTCCATACAA		
GenR-T-F	AGCCCATTCGCCGCCAAGTTCT	480 bp	
GenR-T-R	GCAGCTGTGCTCGACGTTGTCA		
BAR-T-F	TCAGATCTCGGTGACGGGCA	552 bp	
BAR-T-R	ATGAGCCCAGAACGACGCC		
**Generic primers for testing targeted integration**			
RF-1 (HygR constructs)	5′-AAATTTTGTGCTCACCGCCTGGAC	*	T-DNA
RF-2 (HygR constructs)	5′-TCTCCTTGCATGCACCATTCCTTG	*	T-DNA
RF-3 (PgpdA promoter)	5′-TTGCGTCAGTCCAACATTTGTTGCCA	*	T-DNA
RF-7 (GenR constructs)	5′-CTTTGCGCCCTCCCACACAT	*	T-DNA
RF-6 (GenR constructs)	5′-TCAGACACTCTAGTTGTTGACCCCT	*	T-DNA
RF-8 (BarR constructs)	5′-CTGCACTTTTATGCGGTCACACA	*	T-DNA
RF-9 (BarR constructs)	5′-CCTAGGCCACACCTCACCTTATTCT	*	T-DNA

The RF-1 to RF-9 primers anneal inside the different marker genes or introduced promoter and points out into the surrounding genome. These primers should be combined with primers annealing in the genome sequence surrounding the integration site. * The size will depend on the primer design.

### Chemical analysis of the Δ*FaPKS6* strains

The wild type and three Δ*FaPKS6* transformants, a class 2 and two class 3 (replacement), were grown in darkness for 9 days on YES medium at 28°C. Following incubation the metabolites were extracted by means of the micro-scale method [24], using 3:2:1 (ethyl acetate/dichlormethane/methanol 3/2/1 with 1% formic acid). The samples were extracted for 1 h in an ultrasonic bath (Branson™ Bransonic Ultrasonic Cleaner Model 2510), the organic phase was moved to new vials, and evaporated to dryness using N_2_. The samples were resuspended in 500 μl HPLC grade methanol and ultrasonicated for 20 min., filtered through a PFTE filter and analysed by Ultra high performance liquid chromatography-quadruple Time of Flight mass spectrometry (UHPLC-qTofMS).

UHPLC-qTOFMS analysis, of 0.3-2 μL extracts, was conducted on an Agilent 1290 UHPLC equipped with a photo diode array detector scanning 200–640 nm, and coupled to an Agilent 6550 qTOF (Santa Clara, CA, USA) equipped with an electrospray source (ESI). Separation was performed at 60°C and at a flow of 0.35 ml/min on a 2.1 mm ID, 250 mm, 2.7 μm Agilent Poroshell phenyl hexyl column using a water-acetonitrile gradient solvent system containing 20 mM formic acid. The gradient started at 10% acetonitrile and was increased to 100% acetonitrile within 15 min, keeping this for 4 min, returning to 10% acetonitrile in 1 min, and equilibrating for the next sample in 4 min. Samples were analyzed in both ESI^+^ and ESI^−^ scanning m/z 50 to 1700, and with automated data-dependent MS/MS on all major detected peaks, using collision energies of 10, 20 and 40 eV for each MS/MS experiment. A MS/MS exclusion time of 0.04 min was used to get MS/MS of less abounded ions.

Data files were analysed in Masshunter 6.0 (Agilent technologies) in three different ways: i) Aggressive dereplication using lists of elemental composition and the Search by Formula (10 ppm mass accuracy) of all described *Fusarium* metabolites as well as restricted lists of only *F. avenaceum* and closely related species [25]; ii) Searching the acquired MS/MS spectra in an in-house database of approx. 1200 MS/MS spectra of fungal secondary metabolites acquired at 10, 20 and 40 eV [26]; iii) all major UV/Vis and peaks in the BPC chromatograms not assigned to compounds (and not present in the media blank samples) were then also registered. For absolute verification authentic reference standards were available from 130 Fusarium compounds and furthermore 100 have been tentatively identified based on original producing strains, UV/Vis, LogD and MS/MS [25,27].

## Results and discussion

### The USER-Brick vector construction system

The past decade has seen an overwhelming blossom of new cloning systems that allow for easy vector construction, including systems based on Gateway [28], In-Fusion/CloneEZ [29], LIC [30], Gibson assembly [31] and USER cloning. The Uracil Specific Excision Reagent (USER) cloning method dates back to 1991 [32], however, it was initially largely ignored due to the costs associated with synthesising 2-deoxyuridine containing primers and the lack of a proofreading DNA polymerase which would not stall when encountering uracil-containing DNA segments. The technique was revitalized by Nour-Eldin and co-workers [33] based on the identification of the 2-deoxyuridine tolerant Pfu Cx polymerase, and further developed into the restriction enzyme digestion free USER Fusion cloning strategy by Geu-Flores [34]. The great advantage of the USER Fusion strategy compared to classical USER cloning and old-fashioned type II restriction enzyme or nicking enzyme based cloning techniques, is that it allows for scarless fusion of multiple fragments in a single cloning step, and only relies on the presence of an A-N_8–15_-T motif in the junctions (Geu-Flores et al. 2007). Complementary overhangs which allow for directional assembly of multiple fragments is obtained via unique 5′ overhangs on the used primers. The overhangs include a 2-deoxyuridine base which later can be excised and allow for the formation of sticky ends [34,35].

In the past, several USER based vector systems that allow for the construction of vectors compatible with fungal transformations have been presented [15,36]. Although these systems are superior to standard cloning strategies, with respect to ease of experimental design and cloning efficiency, their Achilles heel has always been the dependency on restriction enzyme digestion and nicking of the recipient plasmid. This is due to the dependency of complete digestion of the recipient vector to eliminate false positives which will otherwise shroud the desired correct transformants.

The new USER-Brick system relies entirely on PCR based amplification of all vector elements, which eliminates the time usage, costs and problems associated with the enzymatic digestion of the recipient plasmid. The unique 5′ overhangs introduced into the ends of the individual USER-Bricks during the amplification step ensures directional assembly of all fragments based on standard base pairing rules. The efficacy of the system was tested by constructing 27 different vectors targeting 14 different loci in the genome of *F. avenaceum* and 30 vectors for overexpression of genes from random loci. PCR based testing of 9–10 transformants from each of the 57 constructed vectors, showed that the system displays an assembly fidelity of 97% +/− 0.3 when fusing up to six DNA fragments in a single reaction (Additional file 1: Table S6). In the 16 cases, app. 3%, where the resulting targeted genome modification plasmids did not test positive for the two expected gene specific fragments subsequent PCR tests for the Core-USER bricks in all cases confirmed that they were present.

In the tested setup the gene specific fragments, amplified from genomic DNA, were gel purified to eliminate unspecific products and primer dimers. However, in experiments with up to five fragments unpurified DNA was found to be as efficient as purified DNA, if no unspecific products were detectable by gel electrophoresis. This was not the case for the core USER-Bricks where purification was found to be essential to eliminate the vector DNA that had served as templates for the PCR reactions, which otherwise produced a high rate of false positives.The developed system provides a versatile toolbox that can easily be expanded with additional USER-Bricks, such as alternative selection marker cassettes, promoters and fluorescent reporters, if need be. Either by adding the 5′ overhangs specified in Figure 2 to new bricks with similar functionality as those already used in the system or by designing compatible overhangs for new bricks with novel functionalities.

### Initial ATMT of *F. avenaceum*

The efficiency of ATMT is highly dependent on abiotic factors, such as media composition, temperature and incubation time, and the optimal transformation conditions are typically unique for the individual fungal species [37]. In the case of *F. avenaceum* no protocol for transformation or targeted genome modification had been established, why we set out to develop and optimize a protocol that would allow future analysis of this species via a reverse genetic approach.

To determine the minimum concentration of hygromycin, geneticin and DL-phosphinothricin that could be used in ATMT experiments with *F. avenaceum*, the ATMT process was mimicked without bacterial cells and the growth of the fungus was recorded. The lowest usable hygromycin B concentration was found to be between 75 and 100 μg/ml, why the highest of these were used in subsequent transformation experiments. Similarly for geneticin and DL-phosphinothricin the minimum useful concentrations were found to be 300 μg/ml and 600 μg/ml, respectively.

For the initial transformation experiment random integration of the T-DNA region with the hygromycin resistance cassette, from pRF-HU2, was attempted using ATMT conditions that have previously worked well for *F. graminearum*[12]. The experiments yielded an average transformation frequency of 14 +/−5.1 transformants per 10^6^ *F. avenaceum* spores based on an experiment with five technical replicates. Encouraging results which showed that ATMT based transformation of *F. avenaceum* was possible but would require optimization to yield a useful genetic engineering tool.

### Optimization of ATMT for *F. avenaceum*

#### Pre-induction of A. tumefaciens

**Figure 6:**
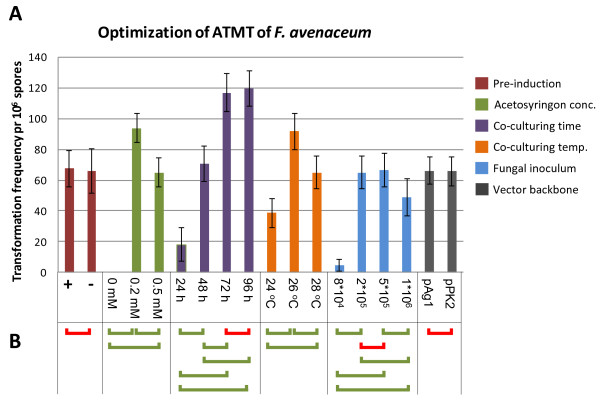
**Results from optimization of the *A. tumefaciens *mediated transformation of *F. avenaceum. *A)** The average number of transformants per 10^6^ spores obtained from three replicates which each included ten agar plates. **B)** Result from Student’s *T*-Test comparing the different incubation conditions: green bars represent statistically significant different results for the two compared conditions, and red bars represent not statistically significant different results (further details can be found in the additional file).

The transformation frequency in ATMT of fungi has previously been shown to be influenced by pre-culturing of the *A. tumefaciens* cells with acetosyringone to induce the virulence response of the bacterium. Examples include *Coccidioides immitis*[38] and *Aspergillus awamori* where as little as 6 h of pre-induction yielded a 10 times increase in the number of transformants [39]. However, the opposite negative effect has also been reported for *Beauveria bassiana*[40]. In the case of *F. avenaceum* pre-induction of the used *A. tumefaciens* strain, for 16 hours, did not result in a statistically significant higher number of colonies (Figure 6). Similar results have been reported for *Aspergillus carbonarius*[41] and *Magnaporthe grisea*[42]. Mullins and co-workers found that pre-induction was not essential for the success of ATMT in the case of *Fusarium oxysporum,* but that the transformation process was delayed compared to experiments including pre-induction [43].

#### Acetosyringone concentration

The presence of a bacterial virulence inducer, such as acetosyringone, during the co-cultivation period, was found to be essential for the successful transformation of *F. avenaceum*. An acetosyringone concentration of 200 μM was found to give the highest number of transformants, while 500 μM resulted in a significant reduction in the number of obtained transformants (Figure 6). This is in contrast to results for *B. bassiana* where an increase in the concentration in the range from 100 μM to 800 μM resulted in increasing numbers of transformants [44]. The observed reduction, for *F. avenaceum*, in the average number of obtained transformants with the higher inducer concentration has also been observed for *Colletotrichum lagenarium* where acetosyringone concentrations above 50 μM resulted in a reduction in transformation frequency [45].

#### Co-culturing time

The co-culturing time was also found to impact the transformation frequency, and a significant increase was observed when the co-culturing time was prolonged from 24 h to 48 h and further to 72 h (Figure 6). Increasing it from 72 h to 96 h did not result in a statistically significant higher number of transformants, suggesting that the *F. avenaceum* mycelium is more susceptible to transformation within the first 72 hours or that the effects of the applied inducer was lost after this time point. A similar effect has been reported for *Cryptococcus neoformans*[46], while Michielse et al. 2004 found a decrease in the transformation frequency for *A. awamori* when extending the co-culturing time from 48 h to 72 h [47]. Compared to *F. graminearum* where the optimal co-culturing time is 48 h, the maximum transformation efficiency for *F. avenaceum* is delayed which is possibly due to a slower germination and growth rate of the latter species, as described by Summerell and co-workers [48].

#### Co-culturing temperature

The temperature at which the co-cultivation was performed also significantly affected the transformation frequency. Incubation at 26°C gave the highest number of transformants while lower, and higher temperatures yielded significantly lower numbers of transformants (Figure 6). This effect has also been observed for other fungal species, such as the ascomycete *Botrytis cinerea*[49], the zygomycete *Mortierella alpine*[50] and the basidiomycete *Hebeloma cylindrosporum*[51]. In all cases, the authors have noted that the effect is linked to the optimal germination/growth temperature of the fungus that is being transformed.

#### Fungal inoculum

Quite interestingly the number of used macroconidia also impacted the number of obtained transformants significantly. The two medium concentrations tested yielded the same number of transformants, while an increase from 5*10^5^ to 1*10^6^ macroconidia per plate lead to a significant reduction (Figure 6). A similar reduction has been reported for *Paecilomyces fumosoroseus*[52]. The biological explanation for the reduction is possibly self-inhibition of germination at higher macroconidia concentrations, as described for other fungal species such as *Colletotrichum fragariae*[53]. The effect of the bacterial inoculum was not tested in the current study, and the concentration was kept constant at an OD_600_ of 0.7 at the stage of co-culturing for all experiments.

#### Binary vector backbone

In addition to the tested abiotic factors, several studies have also shown that the used vector backbone can affect the transformation efficiency. This was not seen for *F. avenaceum* when the two tested vector backbones, pAg1 and pPK2, yielded similar transformation frequencies (Figure 6).

#### ATMT with optimized conditions

The identified optimal conditions differed from the optimal conditions for transformation of *F. graminearum* by requiring a longer co-culturing time (increase from 48 h to 72 h), lower co-culturing temperature (reduction from 28°C to 26°C) and no pre-induction of *A. tumefaciens*. The identified optimal conditions were combined in a new ATMT protocol for *F. avenaceum* and used in a series of new transformation experiments, targeted modification of thirteen different loci, which yielded an average of 140 transformants per 10^6^ transformed spores. This shows that the contribution of the individual parameters to some extent are additive. However part of the expected gain was lost when the optimal conditions were combined, as one would expect the average transformation frequency to reach ~160 per 10^6^ spores based on the experiments where the individual parameters were manipulated. Targeted genome modification of the *FaPKS3* locus using vectors with the geneticin and DL-phosphinothricin selection markers yielded similar transformation frequencies.

### Targeted genome modification in *F. avenaceum* versus *F. graminearum*

To test the difference in targeted integration efficiency for *F. avenaceum* and *F. graminearum*, the orthologous *PKS3/PGL1* locus was chosen as the target. The *PGL1* gene encodes a polyketide synthase that previously has been linked to the formation of the purple-black perithecial pigment of *F. graminearum*[54].

Following transformation, approximately 100 randomly selected transformants from each of the two species were genotyped by PCR. The analysis revealed a targeting efficiency of 85% in *F. graminearum* and 74% in *F. avenaceum* (Table 2). The targeting efficiency in other fungal species has been found to vary greatly, ranging from 0.04% for *Blastomyces dermatitidis*[55] to, the more frequently observed level, 29% observed for *Aspergillus awamori*[39]. Members of the *Fusarium* genus in general display a high targeting efficiency*,* both when transformed by ATMT and protoplast based protocols [56].

**Table 2:** **Results from PCR based genotyping of targeted integration into *F. graminearum *** **and *F. avenaceum***

	**No. tested**	**Class 1 (NHEJ/NHEJ)**	**Class 2 (NHEJ/HR)**	**Class 3 (HR/HR)**	**Gene targeting efficiency (%)**
***F. graminearum PKS3***	104	1	15	88	84.6%
***F. avenaceum PKS3***	100	10	16	74	74.0%
**Other loci in *F. avenaceum***					
**Deletion of 13 PKSs**	129	13	23	93	72.1%
***In locus *** **overexpression 12 PKSs**	116	20	1	95	81.9%

NHEJ = non-homologous end joining; HR = homologous recombination. Class 1 transformants represent random integration events; Class 2 represents transformants where the T-DNA has integration by HR at one end and via an unknown mechanism at the opposite end (likely NHEJ); Class 3 transformants represent the desired gene targeting event where both ends have integrated by HR. The number for ‘other loci’ in *F. avenaceum* represents the average for all 13 deletions and 12 in locus overexpression experiments; see Additional file 1: Table S7 for results for the individual locus.

Genotyping of the transformants from the experiments aimed at targeted deletion or overexpression of thirteen other loci in the *F. avenaceum* genome resulted in comparative targeting efficiencies (deletion: 72.1% and *in locus* overexpression: 81.9%) as found for the *PGL1/PKS3* locus (Table 2 and Additional file 1: Table S7). However, surprisingly, in the twelve experiments aimed at *in locus* overexpression by insertion of a marker gene and promoter between the target gene’s natural promoter and its start site, only ectopic (class 3) and double crossover (class 1) transformants were observed (Table 2 and Additional file 1: Table S7).

Random integration of T-DNA via non-homologous recombination (NHEJ) was more frequent in *F. avenaceum* (10%) than in *F. graminearum* (~1%) (Table 2). The low frequency of random integration in *F. graminearum* concurs well with previous reports by Malz et al. 2006, who found that ATMT based random mutagenesis of *F. graminearum* resulted in 50 times fewer transformants than observed for *F. pseudograminearum*[57]. In the thirty transformations (ectopic overexpression of TFs), which depended on integration via the NHEJ pathways in *F. avenaceum*, significantly fewer transformants were obtained (25 +/−3 per 10^6^ spores) compared to the number of transformants obtained in the above discussed experiments (140 per 10^6^), which depended on HR integrations. This difference is likely due to the fact that integration via the homologous recombination (HR) pathway is not an option due to the lack of homologous recombination sequences in the introduced T-DNA. This supports the initial findings that experimental strategies relying on random mutagenesis by ATMT is feasible in *F. avenaceum*, which is not the case in *F. graminearum*. PCR based genotyping of the obtained transformants showed that 85% (average for all thirty experiments) tested positive for both ends of the TF expression cassette, while 15% showed various levels of truncation (Additional file 1: Table S8).

### Linking of FaPKS6 to production of fusaristatin A

To show that the developed molecular toolkit, USER-Brick system and ATMT of *F. avenaceum*, can be used for functional characterization of genes, we analysed the impact on production of secondary metabolites in the constructed *FaPKS6* deletion strain.

**Figure 7:**
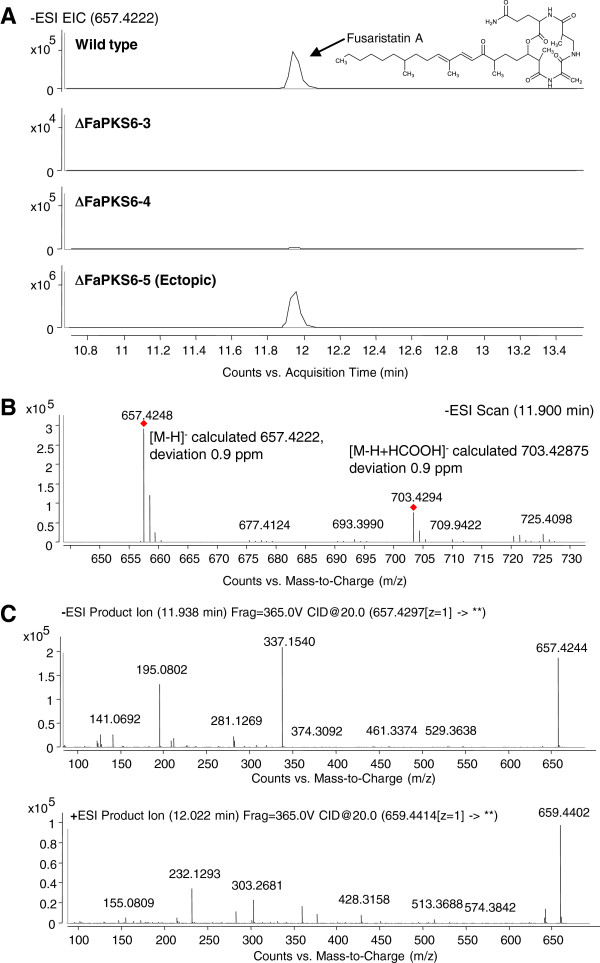
**Linking of *FaPKS6 *to the formation of fusaristatin A. A)** Extracted ion chromatogram, at 657.4222 Scan Frag = 365.0 V, for the wild type and three Δ*FaPKS6* transformants grown on YES medium. The two confirmed *PKS6* replacement strains T3 and T4 have lost the ability to produce fusaristatin A whereas the Δ*FaPKS6*-T5 strain (failed replacement) retains the ability. **B)** Mass spectrum (m/z plot) for the peak at 11.949 minutes and identification of the molecular ion [M-H]^−^ 657.4248 and [M-H + HCOOH]^−^ 703.4294 adduct. **C)** MS/MS based fragmentation in negative and positive mode, using a CID of 20.0 eV: Top panel shows fragmentation of the [M-H]^−^ mass ion and the bottom panel shows fragmentation of the [M + H]^+^ ion.

Chemical profiling of the Δ*FaPKS6* strains compared to the wild type, by UHPLC-qTOFMS, showed that the deletion strain had lost the ability to produce a compound with a retention time of ~11.9 min compared to the wild type (Figure 7A, A). Based on the identification of the [M-H]^−^ and [M + HCOO]^−^ adducts from ESI^−^ and the [M + H]^+^ and [M + Na]^+^ from ESI^+^ elemental compositions could be unambiguously assigned as C_36_H_58_N_4_O_7_. The elemental composition was searched in Antibase2012 [27] and an in-house database [25] showing only one compound with this elemental composition, fusaristatin A (Figure 7A). With the UV/Vis spectrum not providing any structural information, the dereplication was, besides the elemental composition, based on: i) retention time, where fusaristatin A elutes very close to the enniatins (available as reference standards); ii) and MS/HRMS where ammonia was lost in ESI^+^, which is a distinct feature of primary amides (Figure 7C) also the ESI^−^ MS/MS spectrum showed m/z 529.3638 which is consistent with loss of glutamine and m/z 337.1540 with loss of the β-aminoisobutyric acid-glutamine and breakage partial loss of the fatty acid chain at the carbonyl bond. From ESI^+^ MS/MS, the fragments m/z 513.3688 was consistent with loss of glutamine including the oxygen from the fatty acid moiety and m/z 428.3158 with loss of β-aminoisobutyric acid-glutamine including the oxygen from the fatty acid moiety. Finally, the compound was recently identified in the very closely related *F. tricinctum*[58]. Fusaristatin A was first isolated and characterized from the endophytic *Fusarium* sp. YG-45 strain [59], and has later also been identified in the endophyte *Phomopsis longicolla*[60].

The identification is further supported by the domain structure of FaPKS6, and other *Fusarium* PKS6 orthologs, which classifies it as a potential highly reducing PKS with a C-methyltransferase domain. FaPKS6 likely catalyses the formation of the partially reduced decaketide portion of fusaristatin A, which is decorated with three C-bound methyl groups. Following PKS synthesis of the decaketide, it is subsequently elongated by the addition of dehydroalanine, 3-aminoisobutyric acid and glutamine to the carboxylic acid end of the polyketide, likely catalysed by a nonribosomal peptide synthetase (NRPS). A possible candidate for this activity is FaNRPS7 (FA08708) which has three modules and is encoded by a gene located next to the *FaPKS6* gene (FA08709).

This is the first time fusaristatin A has been reported in *F. avenaceum* and the first time its biosynthesis has been linked to a specific polyketide synthase.

Collectively, the linking of *FaPKS6* to fusaristatin A in *F. avenaceum* shows that the developed USER-Brick system and ATMT protocol can be used for efficient functional characterization of genes in the *F. avenaceum* genome.

## Conclusion

The USER-Brick strategy offers reduced experimental costs (no restriction enzymes), easier experimental design, quicker experimental flow, higher cloning efficiency, higher reproducibility between experiments and most importantly, flexibility in the design possibilities. Optimization of the ATMT protocol for *F. avenaceum* yielded an average transformation frequency of 140 transformants per 10^6^ spores in experiments aimed at targeted genome modification. In addition, the optimization process revealed that acetosyringone concentration, co-culturing time and temperature significantly impact the transformation frequency. Combined, the developed USER-Brick system and ATMT protocol offers a generous molecular toolbox that allows for efficient genome engineering in *F. avenaceum* and other filamentous fungi. In the current study, the system was used to successfully link the *FaPKS6* gene to the biosynthesis of fusaristatin A.

## Abbreviations

ATMT: *Agrobacterium tumefaciens* mediated transformation; HR: Homologous recombination; NHEJ: Non-homologous end-joining; PCR: Polymerase chain reaction; PKS: Polyketide synthase encoding gene; T-DNA: Transfer DNA; USER: Uracil specific excision reaction.

## Competing interests

The authors declare that they have no competing interests.

## Authors’ contributions

LQS and PKJ performed the transformation experiments aimed at targeted genome modification of FaPKSs encoding genes. JEL contributed with experiments aimed at ectopic overexpression of TF encoding genes. The metabolomics profiling and mass spectrometry based identification of Fusaristatin A was performed by KFN. RJNF conceived the study, developed the USER-Brick platform, optimized ATMT of *F. avenaceum*, wrote and edited the manuscript. All authors read and approved the final manuscript.

## Supplementary Material

Additional file 1 **Figure S1.** Pre-induction of *A. tumefaciens* has no effect on the transformation frequency. **Figure S2.** The effect of three different acetosyringone concentrations in the co-culturing medium. **Figure S3.** Increasing the co-culturing time to 72 h result in increased numbers of transformants. **Figure S4.** The optimal co-culturing temperature when transforming *F. avenaceum* is 26°C. **Figure S5.** The optimal number of macroconidia per transformation plate was found to be between 2*10^5 and 5*10^5. **Figure S6.** The vector backbone was not found to affect the transformation frequency. **Table S1.** List of USER-Bricks needed for different types of vector constructs. **Table S2.** Gene specific primers for vector construction: deletion and *in locus* overexpression. **Table S3.** Gene specific primers for vector construction: Ectopic overexpression of PKS associated transcription factors. **Table S4.** Primers for validation of genomic modifications in *F. avenaceum* and *F. graminearum*. **Table S5.** Primers for validation of genomic modifications in *F. avenaceum*. **Table S6.** Vector construction efficiency with the USER-Brick approach. **Table S7.** Results from PCR based genotyping of the constructed *F. avenaceum* PKS deletion and *in locus* overexpression strains. **Table S8.** Results from PCR based genotyping of the constructed *F. avenaceum* strains expressing PKS associated transcription factors from random loci in the genome. Click here for file
