# Supplementary material for: Genetic transformation of Fusarium avenaceum by Agrobacterium tumefaciens mediated transformation and the development of a USER-Brick vector construction system
Source: BMC Mol Biol. 2014 Jul 22;15:15. doi: 10.1186/1471-2199-15-15 (PMC4133957; doi:10.1186/1471-2199-15-15)
Supplement: Additional file 1 — Figure S1. Pre-induction of A. tumefaciens has no effect on the transformation frequency. Figure S2. The effect of three different acetosyringone concentrations in the co-culturing medium. Figure S3. Increasing the co-culturing time to 72 h result in increased numbers of transformants. Figure S4. The optimal co-culturing temperature when transforming F. avenaceum is 26°C. Figure S5. The optimal number of macroconidia per transformation plate was found to be between 2*10^5 and 5*10^5. Figure S6. The vector backbone was not found to affect the transformation frequency. Table S1. List of USER-Bricks needed for different types of vector constructs. Table S2. Gene specific primers for vector construction: deletion and in locus overexpression. Table S3. Gene specific primers for vector construction: Ectopic overexpression of PKS associated transcription factors. Table S4. Primers for validation of genomic modifications in F. avenaceum and F. graminearum. Table S5. Primers for validation of genomic modifications in F. avenaceum. Table S6. Vector construction efficiency with the USER-Brick approach. Table S7. Results from PCR based genotyping of the constructed F. avenaceum PKS deletion and in locus overexpression strains. Table S8. Results from PCR based genotyping of the constructed F. avenaceum strains expressing PKS associated transcription factors from random loci in the genome. [file 1471-2199-15-15-S1.docx]

**Additional file for “Genetic transformation of *Fusarium avenaceum* by *Agrobacterium tumefaciens* mediated transformation and the development of a USER-Brick vector construction system”**

By Lisette Quaade Sørensen, Jesper Erup Larsen, Paiman Khorsand-Jamal, Kristian Fog Nielsen and Rasmus John Normand Frandsen

Plots showing the number of obtained transformants with the different tested ATMT conditions and results from Student’s T-Test comparing the different conditions:

**Figure S1:** Pre-induction of the used *A. tumefaciens* cells before co-culturing with *F. avenaceum* spores. Pre-incubation has no effect on the transformation frequency (P = 0.634)

**Figure S2:** The effect of three different acetosyringone concentrations in the co-culturing medium. A concentration of 0.2 mM and 0.5 mM gives significantly more colonies than 0 mM (P = 1.22 E-30 and P = 3.95 E-25). And 0.2 mM gives significantly more colonies than 0.5 mM (P = 1.06 E-16).

**Figure S3:** Increasing the co-culturing time to 72 h result in increased numbers of transformants, while additionally increasing the time did not yield significantly more transformants.

| 48 h co-cultivation gives significantly more colonies than 24 h | P = 9.84 E-25 |
| --- | --- |
| 72 h co-cultivation gives significantly more colonies than 24 h | P = 9.41 E-38 |
| 96 h co-cultivation gives significantly more colonies than 24 h | P = 1.23 E-39 |
| 72 h co-cultivation gives significantly more colonies than 48 h | P = 3.22 E-21 |
| 96 h co-cultivation gives significantly more colonies than 48 h | P = 2.33 E-23 |
| 96 h co-cultivation does **NOT** give more colonies than 72 h | P = 0.372 |

**Figure S4:** The optimal co-culturing temperature when transforming *F. avenaceum* is 26 ^o^C

| 26 oC gives significantly more colonies than 24 ^o^C | P = 6.18 E-26 |
| --- | --- |
| 28 oC gives significantly more colonies than 24 ^o^C | P = 6.83 E-14 |
| 26 oC gives significantly more colonies than 28 ^o^C | P = 6.5 E-13 |

**Figure S5:** The optimal number of macroconidia per transformation plate was found to be between 2*10^5 and 5*10^5.

| 2*10^5 give significantly more colonies than 8*10^4 | P = 4.74 E-27 |
| --- | --- |
| 5*10^5 give significantly more colonies than 8*10^4 | P = 8.99 E-25 |
| 1*10^6 give significantly more colonies than 8*10^4 | P = 1.31 E-18 |
| 2*10^5 does NOT give statistically significantly fewer colonies than 5*10^5 | P = 0.586 |
| 2*10^5 gives significantly more colonies than 1*10^6 | P = 2.68 E-06 |
| 5*10^5 gives significantly more colonies than 1*10^6 | P = 7.98 E-07 |

**Figure S6:** The vector backbone was not found to affect the transformation frequency.

| pAg1 result in more transformants than pPK2 |  | P = 0,891 |
| --- | --- | --- |

**Table S1:** List of USER-Bricks needed for different types of vector constructs.

| **Purpose** | **Fragments** | **Fig.** |
| --- | --- | --- |
| **Targeted replacement/Deletion** | | |
|  | B1 | 2A |
|  | B2 |  |
|  | HygR1^A^ |  |
|  | Gene-X HRS1 (Before gene) |  |
|  | Gene-X HRS2 (After gene) |  |
| ***In locus* overexpression** | | |
|  | B1 | 2B |
|  | B2 |  |
|  | HygR1^A^ |  |
|  | PgpdA^B^ |  |
|  | Gene-X HRS1 (Before gene) |  |
|  | Gene-X HRS3 (Start of CDS) |  |
| **Expression from random (ectopic) locus with the genes own promoter** | | |
|  | B1 | 2C |
|  | B2e |  |
|  | HygR1^A^ |  |
|  | Gene-X (promoter + CDS + terminator) |  |
| **Expression from random (ectopic) locus with ’artifical’ promoter** | | |
|  | B1 | 2D |
|  | B2e |  |
|  | HygR1^A^ |  |
|  | PgpdA^B^ |  |
|  | Gene-X (promoter + CDS + terminator) |  |
| **Expression from fixed (ectopic) locus with ‘artifical’ promoter** | | |
|  | **B1** | 3A |
|  | **B2** |  |
|  | HygR1^A^ |  |
|  | PgpdA^B^ |  |
|  | **CDSf (gene to be expressed)** |  |
|  | **HRS1 (fixed locus)** |  |
|  | **HRS2f (fixed locus)** |  |
| **Transcriptional reporter construct expressed from random (ectopic) locus** | | |
|  | B1 | 3B |
|  | B2e |  |
|  | HygR1^A^ |  |
|  | PgpdA^B^ |  |
|  | PromoterX (promoter to be analyzed) |  |
|  | **mRFP-2** |  |

**Table S1 Continues**

| **Purpose** | **Fragments** | **Fig.** |
| --- | --- | --- |
| **Transcriptional reporter construct expressed from fixed locus** | | |
|  | B1 | 3C |
|  | B2 |  |
|  | HygR1^A^ |  |
|  | PromoterX (promoter to be analyzed) |  |
|  | **mRFP-1** |  |
|  | **HRS1 (fixed locus)** |  |
|  | **HRS2f (fixed locus)** |  |
| **N-terminal mRFP tagging expressed with the genes natural promoter and locus** | | |
|  | **B1** | 3D |
|  | B2 |  |
|  | HRS1 |  |
|  | HRS3f |  |
|  | HygR1^A^ |  |
|  | PromoterX (promoter to be analyzed) |  |
|  | **mRFP-3** |  |

**Table S2:** Gene specific primers for vector construction (deletions and in locus overexpression)

| **Gene** | **Primer name** | **Sequence (5’ to 3’)** | **Product bp** |
| --- | --- | --- | --- |
| FaPKS3  (FA03082.2) | FaPKS3-U1  FaPKS3-U2 | AGGTCGTATUGGGAGTGAAGAAGTCGTATTCGAC  AGTATTGCGUGACAAAGGTGTTGAATCTTTTGGG | 1536 |
|  | FaPKS3-U3  FaPKS3-U4 | ATGACCTAGUAAGAATTGGCAGTGATGGCCCG  ATTAAACCTUAACTCCCTGTGTCCGAGATATTCG | 1534 |
| F**g**PKS3  (FGSG_09182) | FgPKS3-U1  FgPKS3-U2 | AGGTCGTATUTGTGTTACCCCATCGCTGAGTG  AGTATTGCGUGGGAATAAGGTCTGCTGTGAAA | 1543 |
|  | FgPKS3-U3  FgPKS3-U4 | ATGACCTAGUGGAGTAGGATATAGCGCAGGTA  ATTAAACCTUGAAGGTGCGTTTAGGTGACAGAA | 1566 |
| FaPKS5  (FA11674.2) | FaPKS5-U1  FaPKS5-U2 | AGGTCGTATUATTTGGAGCATGTCGCGGC  AGTATTGCGUAGTTGGAGACAAAAGGTGCTGC | 1525 |
|  | FaPKS5-U3  FaPKS5-U4 | ATGACCTAGUATGTGTAGGAGGAAAGTCGATTCG  ATTAAACCTUCTTGCAACACATCCTCATCCATCC | 1550 |
|  | FaPKS5-O3  FaPKS5-O4 | AGGCTGTAUGAGCCCTCCAGCCCCGATCGCCATCA  ATTAAACCTUGGATCTGCTAGCATGTTAGAGCGA | 1521 |
| FaPKS15  (FA12691.2) | FaPKS15-U1  FaPKS15-U2 | AGGTCGTATUGGTGAACTGCGTACCCATTGACC  AGTATTGCGUTGTGACTCTCGGCCTTTCCGATG | 1522 |
|  | FaPKS15-U3  FaPKS15-U4 | ATGACCTAGUTGAGGACAAATGATGGAATACCACC  ATTAAACCTUGCGCCATGCTAGTCGGTTTACTT | 1554 |
|  | FaPKS15-O3  FaPKS15-O4 | AGGCTGTAUGTCCGGACCAAGTACGGAGAATCAAAGA  ATTAAACCTUTCAAAAGAGCCAGCTGCCTCGAG | 1542 |
| FaPKS27  (FA10284.2) | FaPKS27-U1  FaPKS27-U2 | AGGTCGTATUGTTTGTGAATGCTCGGATGTCGG  AGTATTGCGUTGTGGACAAGAGATTGGGGTTATAG | 1542 |
|  | FaPKS27-U3  FaPKS27-U4 | ATGACCTAGUTAGTGCTTGTGTTATTGTAAATA  ATTAAACCTUGAGCCGCTATTCGAGGATGTATG | 1552 |
|  | FaPKS27-O3  FaPKS27-O4 | AGGCTGTAUGCCTTCTCCCGGAGAGCCCATCG  ATTAAACCTUCGCGGTTCTCGCCAGAGAAGAAAGC | 1557 |
| FaPKS37  (FA04649.2) | FaPKS37-U1  FaPKS37-U2 | AGGTCGTATUACAGAAAGAACGAGGCGGTGGTT  AGTATTGCGUGTTGAATGTTCAAGGTAGTTGTCAGAAA | 1524 |
|  | FaPKS37-U3  FaPKS37-U4 | ATGACCTAGUTAGGTGGTTGGTTTTGATGGAGC  ATTAAACCTUCCCTAGCTGCACTTCTGGTATATGG | 1520 |
|  | FaPKS37-O3  FaPKS37-O4 | AGGCTGTAUGTCTTCTTCAAGAATATACGTCT  ATTAAACCTUTCATTGCTTGTGACACCATAGTACACG | 1557 |
| FaPKS38 (FA04674.2) | FaPKS38-U1  FaPKS38-U2 | AGGTCGTATUGTCGCAGAGGGTTGTTTCCAAAAG  AGTATTGCGUAATAGGTGATGAATGCGAGTGCA | 1537 |
|  | FaPKS38-U3  FaPKS38-U4 | ATGACCTAGUTGATGCTGCTGGTTGCATAGTGAC  ATTAAACCTUTCACCCAAAGATATCCCACCGCG | 1541 |
|  | FaPKS38-O3  FaPKS38-O4 | AGGCTGTAUGTCCTCCACCAACACCCAAGAAG  ATTAAACCTUACGGAGCTCAAAGCAGCAATATCG | 1519 |
| FaPKS41  (FA07658.2) | FaPKS41-U1  FaPKS41-U2 | AGGTCGTATUCTGTCTTCAACTCGATTCGCCAC  AGTATTGCGUTGGAACGAGTTGAGTGTTCCAGT | 1520 |
|  | FaPKS41-U3  FaPKS41-U4 | ATGACCTAGUTAGAGCTTGGGGTTTTCATCACTATAC  ATTAAACCTUCCGGTGAGCATGGCGTTATATATC | 1535 |
|  | FaPKS41-O3  FaPKS41-O4 | AGGCTGTAUGGACTCAAAACCTAGTGAGCCTATTGCCG  ATTAAACCTUTGCCAGATCCCTCAAAGAAGCTTG | 1558 |
| FaPKS42  (FA07835.2) | FaPKS42-U1  FaPKS42-U2 | AGGTCGTATUTTAGGATCACGAGAGGGTGACATG  AGTATTGCGUGATGTAAAGGTTGACAACGGATCTG | 1520 |
|  | FaPKS42-U3  FaPKS42-U4 | ATGACCTAGUTGAGAGCATAAAAACATATAGTGT  ATTAAACCTUACATTATTCAGGTTACCATGGAT | 1520 |
|  | FaPKS42-O3  FaPKS42-O4 | AGGCTGTAUGCCTTTGTTCACCACTGATATGTGCC  ATTAAACCTUGCGCTCGAATGTTGACTTGATCTC | 1557 |
| FaPKS43  (FA07921.2) | FaPKS43-U1  FaPKS43-U2 | AGGTCGTATUGCCTTCAACTCCAGATGACAGGA  AGTATTGCGUTTTGGTGAGGATTGCAGAATCTG | 1539 |
|  | FaPKS43-U3  FaPKS43-U4 | ATGACCTAGUTGATTCTAATGTAGTGTGGAGTT  ATTAAACCTUTTTCTGGGCACATCATACCAATG | 1553 |
|  | FaPKS43-O3  FaPKS43-O4 | AGGCTGTAUGACACAAACAGAAAGCCGTATTGC  ATTAAACCTUGCCACTGCCTTGTGTTGGAGATT | 1530 |
| FaPKS44  (FA11699.2) | FaPKS44-U1  FaPKS44-U2 | AGGTCGTATUCTTCTCAGCTCTCTGGTACAGGT  AGTATTGCGUATTTACAACAGTTGTGTTGTCTG | 1558 |
|  | FaPKS44-U3  FaPKS44-U4 | ATGACCTAGUTAACCTCCTCCTTTATAGCTAAG  ATTAAACCTUTAAGTGACTACAGAAATAATGCA | 1520 |
|  | FaPKS44-O3  FaPKS44-O4 | AGGCTGTAUGACCAGCAGAGACAGTTGGAAGAC  ATTAAACCTUGCTTCTGGGGAGAAACTGCATCT | 1527 |
| FaPKS45  (FA08391.2) | FaPKS45-U1  FaPKS45-U2 | AGGTCGTATUCAAATGGGCTCTATTCGACACAGC  AGTATTGCGUAATGGCGGATATGTGAGGCTTGC | 1520 |
|  | FaPKS45-U3  FaPKS45-U4 | ATGACCTAGUTGATAATTTGATCGACAGCTAACTTGA  ATTAAACCTUTATGACCTCTCCTGTAAATTGCACTCA | 1520 |
|  | FaPKS45-O3  FaPKS45-O4 | AGGCTGTAUGGGCAGTGCCAACGAGCCTATCG  ATTAAACCTUGACAGCTGAACACGAGCCTCCGT | 1538 |
| FaPKS47  (FA08496.2) | FaPKS47-U1  FaPKS47-U2 | AGGTCGTATUGCGTACACAACAACTGCGACTATG  AGTATTGCGUTGAGTTGTGTCAAACTATAGACTCGGA | 1525 |
|  | FaPKS47-U3  FaPKS47-U4 | ATGACCTAGUTGAGGTCCACCCAGAGTTATTTTAGT  ATTAAACCTUGTCAGGATTATTGAGCGCATCGAC | 1521 |
|  | FaPKS47-O3  FaPKS47-O4 | AGGCTGTAUGATTTCTAAACCAGAGCCTATTGCTATT  ATTAAACCTUTTCAATTGAGCAATCAAGGAGGC | 1519 |
| FaPKS48  (FA10226.2) | FaPKS48-U1  FaPKS48-U2 | AGGTCGTATUCCTGCTTCCAAAGAGCGACTTCT  AGTATTGCGUCTTTGGATAATCTTGCTTCATTTGTCT | 1521 |
|  | FaPKS48-U3  FaPKS48-U4 | ATGACCTAGUTAAGGGTTATTCCGCAGGGCATT  ATTAAACCTUCTAACCAGGCGCTTAGAATACTCG | 1545 |
|  | FaPKS48-O3  FaPKS48-O4 | AGGCTGTAUGCATCCGACAAAGGTCTTCATTT  ATTAAACCTUAGTCAACAAGGCCAGTCGTTGGG | 1530 |
| FaPKS6  (FA08709.2) | FaPKS6-U1  FaPKS6-U2 | AGGTCGTATUAAATATTCACTATTTCGTCTGAG  AGTATTGCGUTGTGTGTAAAAGATGAATATTTT | 1604 |
|  | FaPKS6-U3  FaPKS6-U4 | ATGACCTAGUTGAACAAGTAGCTGTATCTAGGGGCG  ATTAAACCTUCTGTCATGAGATTGCGCAGCCAG | 1520 |

**Table S3:** Gene specific primers for vector construction. Ectopic overexpression of PKS associated transcription factors.

| **Gene** | **Primer name** | **Sequence (5’ to 3’)** | **Product (bp)** |
| --- | --- | --- | --- |
| FaPKS5-TF1  (FA11665.2) | FaPKS5-TF1-F | AGGCTGTAUGAAACGTTCAGCTGCGCA | 2945 |
|  | FaPKS5-TF1-R | ATTAAACCTUTGACCTTTGCGAGATGGATCTATAT |  |
| FaPKS7-TF1  (FA07185.2) | FaPKS7-TF1-F | AGGCTGTAUGGCGGCCACTGAGCAGAA | 3437 |
|  | FaPKS7-TF1-R | ATTAAACCTUATCGAGCTTGAAACAACGACCC |  |
| FaPKS8-TF1  (FA02817.2) | FaPKS8-TF1-F | AGGCTGTAUGACATCAACAAAACCGACGTG | 1250 |
|  | FaPKS8-TF1-R | ATTAAACCTUTGAGCTTGCGTAGATTCATAAGGAC |  |
| FaPKS8-TF2  (FA02822.2) | FaPKS8-TF2-F | AGGCTGTAUGTCGAATCATGGTAGGAAAAGAGG | 5221 |
|  | FaPKS8-TF2-R | ATTAAACCTUGCGAACTTCATGCACCAATATTC |  |
| FaPKS8-TF3  (FA02831.2) | FaPKS8-TF3-F | AGGCTGTAUGGATGAGAACGAGATAACTGTCCA | 2539 |
|  | FaPKS8-TF3-R | ATTAAACCTUATTAACCAGTTGTGTGCGGGTG |  |
| FaPK11-TF1  (FA04705.2) | FaPKS11-TF1-F | AGGCTGTAUGTATCCTGCTGTGCCGCC | 2852 |
|  | FaPKS11-TF1-R | ATTAAACCTUCCGAACAAATGGTGGTCGAA |  |
| FaPKS11-TF2  (FA04710.2) | FaPKS11-TF2-F | AGGCTGTAUGAGCCTAGCAGCTCACGATATAC | 1596 |
|  | FaPKS11-TF2-R | ATTAAACCTUATAGAGATTCTGAGCGGTGGCG |  |
| FaPKS11-TF3  (FA04714.2) | FaPKS11-TF3-F | AGGCTGTAUGTCAAGTCCAAGGTTTCACGA | 2620 |
|  | FaPKS11-TF3-R | ATTAAACCTUTGTACAGAGATTTAGAAACCTTGGGC |  |
| FaPKS15-TF1  (FA12686.2) | FaPKS15-TF1-F | AGGCTGTAUGAGCTTTAATCGACAAAG | 2487 |
|  | FaPKS15-TF1-R | ATTAAACCTUCTGAAGTTCAACGTTCCTTG |  |
| FaPKS26-TF1  (FA02207.2) | FaPKS26-TF1-F | AGGCTGTAUGAAAACACGGACCGGCAA | 3379 |
|  | FaPKS26-TF1-R | ATTAAACCTUGCGTTAATATTATGCTTGGCGG |  |
| FaPKS27-TF1  (FA10277.2) | FaPKS27-TF1-F | AGGCTGTAUGGATCCAGGTCCGGCATA | 2601 |
|  | FaPKS27-TF1-R | ATTAAACCTUCATGATGTTACTGGCAGGATCTACTG |  |
| FaPKS27-TF2  (FA10291.2B_RF) | FaPKS27-TF2-F | AGGCTGTAUGGCGACCATACAGGGCGT | 1552 |
|  | FaPKS27-TF2-R | ATTAAACCTUAGCTTACTAGCTATTAATAT |  |
| FaPKS27-TF3  (FA10292.2) | FaPKS27-TF3-F | AGGCTGTAUGACAGGCCTCTATTCCCTTCC | 2909 |
|  | FaPKS27-TF3-R | ATTAAACCTUGTTCTCATCAGTTTGACGTATCTTGC |  |
| FaPKS38-TF1  (FA04676.2) | FaPKS38-TF1-F | AGGCTGTAUGGATCTCGGTCCACCATGG | 2760 |
|  | FaPKS38-TF1-R | ATTAAACCTUCTCACGATGTCTCCGCATCAC |  |
| FaPKS40-TF1  (FA07763.2) | FaPKS40-TF1-F | AGGCTGTAUGGTTGGAAGTCAGATTAATGATCC | 2315 |
|  | FaPKS40-TF1-R | ATTAAACCTUCTTTACCGTCAATTTCATTATCCCG |  |
| FaPKS40-TF2  (FA07780.2) | FaPKS40-TF2-F | AGGCTGTAUGTCTAAAAACCCGACAAATCAAAG | 2068 |
|  | FaPKS40-TF2-R | ATTAAACCTUTTCAAACCGACGAACGCTTTCAA |  |
| FaPKS41-TF1  (FA07646.2) | FaPKS41-TF1-F | AGGCTGTAUGGAGCCCTCGGAGCAACC | 2337 |
|  | FaPKS41-TF1-R | ATTAAACCTUACAAAAGAAACACGTGCCGTAGA |  |
| FaPKS42-TF1  (FA07842.2) | FaPKS42-TF1-F | AGGCTGTAUGATGGACGCAGCAGGTTTG | 1317 |
|  | FaPKS42-TF1-R | ATTAAACCTUCCCTTATTCTCTTCTCTAGGCCTTTG |  |
| FaPKS42-TF2  (FA07844.2) | FaPKS42-TF2-F | AGGCTGTAUGGCCGAAACAAATGCCAG | 2189 |
|  | FaPKS42-TF2-R | ATTAAACCTUAGATGGTGGTGAGTGTGAGTTTGA |  |
| FaPKS42-TF3  (FA07848.2) | FaPKS42-TF3-F | AGGCTGTAUGCTTTGCCTAGCATACCAATATGG | 1735 |
|  | FaPKS42-TF3-R | ATTAAACCTUGCAACAGTACGTTGTTAATCCAACC |  |
| FaPKS43-TF1  (FA07914.2) | FaPKS43-TF1-F | AGGCTGTAUGCAGCGCCTGTTCTCATC | 2698 |
|  | FaPKS43-TF1-R | ATTAAACCTUGGGAAGCTTAGAGCAAAGTTTGG |  |
| FaPKS44-TF1  (FA11693.2) | FaPKS44-TF1-F | AGGCTGTAUGGCACGCAGGGCTCATAA | 1764 |
|  | FaPKS44-TF1-R | ATTAAACCTUTATTTCTAATATGACTGTGT |  |
| FaPKS44-TF2  (FA11694.2) | FaPKS44-TF2-F | AGGCTGTAUGTACTCAATATCTGAACCTGCC | 2024 |
|  | FaPKS44-TF2-R | ATTAAACCTUACAAACAATGCGGGTGTAATGAC |  |
| FaPKS46-TF1  (FA11712.2) | FaPKS46-TF1-F | AGGCTGTAUGCCTCGTCCCAAAGTTCG | 2943 |
|  | FaPKS46-TF1-R | ATTAAACCTUTCGTTCTATTTGGGCGAAGCA |  |
| FaPKS45-TF1  (FA08397.2) | FaPKS45-TF1-F | AGGCTGTAUGTCAGCAACCGAAGCCAG | 2478 |
|  | FaPKS45-TF1-R | ATTAAACCTUTAGCGAGACAGCTTTGCGAAA |  |
| FaPKS49-TF1  (FA08722.2) | FaPKS49-TF1-F | AGGCTGTAUGCACAGAGCCAAACGTAAAAG | 2669 |
|  | FaPKS49-TF1-R | ATTAAACCTUTCCAGTAAGACGACAAGGTCATTTT |  |
| FaPKS47-TF1  (FA08504.2) | FaPKS47-TF1-F | AGGCTGTAUGCAAATTAGCGTGAGTCGGC | 1924 |
|  | FaPKS47-TF1-R | ATTAAACCTUCGAATCTGAAGGGAGAAGTAGACATT |  |
| FaPKS47-TF2  (FA08507.2) | FaPKS47-TF2-F | AGGCTGTAUGCAGCCCCAAGCTCAAAT | 2644 |
|  | FaPKS47-TF2-R | ATTAAACCTUTACATCGCTGCTGATGAGGCA |  |
| FaPKS48-TF1  (FA10220.2) | FaPKS48-TF1-F | AGGCTGTAUGGCAAACATATCAACTCCAGATC | 2949 |
|  | FaPKS48-TF1-R | ATTAAACCTUAGAAGCATGGTGCGAAAGATCC |  |
| FaPKS48-TF2  (FA10221.2) | FaPKS48-TF2-F | AGGCTGTAUGGACACCGCCAATAACAACTG | 1767 |
|  | FaPKS48-TF2-R | ATTAAACCTUACAGCCCTAGTCCGATCCACA |  |

**Table S4:** Primers for validation of genomic modifications in *F. avenaceum* and *F. graminearum*. T3 primers were combined with the RF-1 primer, T4 with RF-2 and T5 with RF-5.

| **Gene** | **Primer name** | **Sequence (5’ to 3’)** | **Product (bp)** |
| --- | --- | --- | --- |
| Standard test  primers | RF-1 | AAATTTTGTGCTCACCGCCTGGAC | Combined T3 |
|  | RF-2 | TCTCCTTGCATGCACCATTCCTTG | Combined T4 |
|  | RF-3 | TTGCGTCAGTCCAACATTTGTTGCCA | Combined T5 |
| FaPKS3  (FA03082.2) | FaPKS3-T1  FaPKS3-T2 | GGCATTGTTCCCACTGCTGT  CACTGCCGTCTAGAGGACAA | 445 |
|  | FaPKS3-T3  FaPKS3-T4 | CGGATTATGCTCTCGCCTTGAG  AGAGCGCTCCACTCCTCAACCT | 2032  1902 |
| F**g**PKS3  (FGSG_09182) | FgPKS3-T1  FgPKS3-T2 | TGATGGCACTGTAGTTGAGG  GTGGTGCTCTCAAGTTTGGG | 459 |
|  | FgPKS3-T3  FgPKS3-T4 | TACTTCGATGAATGAGTGACCCTCT  TGGATGGGCTCTGGGAGGTCTT | 1924  2001 |
| FaPKS5  (FA11674.2) | FaPKS5-T1  FaPKS5-T2 | GTTTCCAAAGCTGTGCCCTTG  GGTTCCACAGACTTTTGATCAGAGG | 506 |
|  | FaPKS5-T3  FaPKS5-T4 | GGTGCATCTCGTCATGGGTAGG  GCGTCCACATAGCTCTCTTCAACG | 1805  1895 |
|  | FaPKS5-T5 | TCGTACTCCCGTTAAGCTGGTG | 1903 |
| FaPKS15  (FA12691.2) | FaPKS15-T1  FaPKS15-T2 | GTGATTGACACGGCTTGTTCG  GATCGCCTTTCTGAGTGCCA | 451 |
|  | FaPKS15-T3  FaPKS15-T4 | GGAAAATATTCTGACTTGGGACCTCTC  TAAAACGAAAGAATAAAACATTATCTGTCC | 1862  1862 |
|  | FaPKS15-T5 | TCTCTGAGGATGATGGACTGAAATAAC | 1908 |
| FaPKS27  (FA10284.2) | FaPKS27-T1  FaPKS27-T2 | CCCAACTGCGCAACCTGAGCAT  CCTTGGCCTGTTGGATCGTTC | 465 |
|  | FaPKS27-T3  FaPKS27-T4 | GCTCAGTGGAACGAATCAAAAGCT  TTTCCGCTCCCTTCGCAATA | 1917  2000 |
|  | FaPKS27-T5 | ATTTTCCTACCCATAGACGCCC | 1955 |
| FaPKS37  (FA04649.2) | FaPKS37-T1  FaPKS37-T2 | AAAGCAGTGACTCTGAAACGCC  CGATGATGGACTTGACCTTTGGG | 454 |
|  | FaPKS37-T3  FaPKS37-T4 | GGATCTTGTACTGGTGGGGCAA  CGACAGCAGCTCCTTTCTATCATG | 1945  1991 |
|  | FaPKS37-T5 | ACGTTTGTTCCGCCGGCAAT | 2062 |
| FaPKS38  (FA04674.2) | FaPKS38-T1  FaPKS38-T2 | ATCTTGCGGGCGCAAATGTCACTAC  TTCAGAGCCGAGGACCTTAGCG | 464 |
|  | FaPKS38-T3  FaPKS38-T4 | GGATGGCGGGCGTTGTTAAA  ACCACAAGTGGGTGATTTGTAGTTGAACCA | 1907  1996 |
|  | FaPKS38-T5 | TGAGCACCCTGGCCAGTAAA | 1958 |
| FaPKS41  (FA07658.2) | FaPKS41-T1  FaPKS41-T2 | CCTACGTTCATTGCATGTGCC  TTATCGACGGTGTAGCGGCC | 459 |
|  | FaPKS41-T3  FaPKS41-T4 | TCCATCCGGAATCGCCTTGG  GACAACTTTGACATTGACGATAGAGTCG | 1905  1995 |
|  | FaPKS41-T5 | CGTCGTCGAGAGCACTTTTGA | 1902 |
| FaPKS42  (FA07835.2) | FaPKS42-T1  FaPKS42-T2 | GGATCTCAAGATCATCTCGCGC  CCGTTCTGGTTGACAGGCAG | 450 |
|  | FaPKS42-T3  FaPKS42-T4 | GCACTATAGCATGGGAGATCTTACGAC  TTCTGAGGAGCACCTCGACTGC | 1916  1999 |
|  | FaPKS42-T5 | GCATGGGCGTCAAACAACTG | 1950 |
| FaPKS43  (FA07921.2) | FaPKS43-T1  FaPKS43-T2 | ATGACGCTAACAACAAGATGACCTACC  AAAGAGAGCATGGAAGCCACAAT | 454 |
|  | FaPKS43-T3  FaPKS43-T4 | TGATCCCTGCACGGACGTCT  AACAACAGGCTGTAGCTGCTCTCC | 1916  1993 |
|  | FaPKS43-T5 | CTCCAAGAGCTGCATCGAGTTC | 1903 |
| FaPKS44  (FA11699.2) | FaPKS44-T1  FaPKS44-T2 | AGCAGGTCACTGTCGTAACGAAG  CGAGATAGAGACGAGAAGAGGATGAAGA | 463 |
|  | FaPKS44-T3  FaPKS44-T4 | TGCGACAATGACAGCCGACA  CGCTAGTTCCAAGATACTTGAGTTTGC | 1857  1867 |
|  | FaPKS44-T5 | TGGGAAAATGCTCGCGTCGA | 1918 |
| FaPKS45  (FA08391.2) | FaPKS45-T1  FaPKS45-T2 | ACAAGGCGACAGGACAGATCAC  TTGCATTGGCCACCATTGAC | 450 |
|  | FaPKS45-T3  FaPKS45-T4 | CCACAGTGTTTGAGATACGGTTGG  AATGTATACACATGGGCCCTGACG | 1892  1895 |
|  | FaPKS45-T5 | AAGATGATGACGCAAAAGCGAC | 1953 |
| FaPKS47  (FA08496.2) | FaPKS47-T1  FaPKS47-T2 | ACGCCATCGGCCAAAACCTG  GGGAGTGGCCACAACAACAATATC | 461 |
|  | FaPKS47-T3  FaPKS47-T4 | GCTTTGATGTTCGGATTGTGTGG  TGGCAAGCATGTCCTCCTCG | 1856  1999 |
|  | FaPKS47-T5 | TCGATCTTGGAGACTAACTCGGG | 1904 |
| FaPKS48  (FA10226.2) | FaPKS48-T1  FaPKS48-T2 | GCAGATGGCTCAGCAAGACAA  TCACGAAGGTCTGACTGCATGA | 453 |
|  | FaPKS48-T3  FaPKS48-T4 | CCTCCTGTCGATAAGATTGAGGCTT  ACTCTGTCGCTAATTTGGTGGTTAGG | 1864  1994 |
|  | FaPKS48-T5 | GAAGTAGTTGATCCTGCCAGGGA | 1960 |
| FaPKS6  (FA08709.2) | FaPKS6-T1  FaPKS6-T2 | GGCATGTGGAAGCTCGTGGGAT  GCCGTTGCTTCAAGGAGTCG | 452 |
|  | FaPKS6-T3  FaPKS6-T4 | CAGCAACATGCGAGATGGGTC  GGATATGGTAACGACCAAAGTGAACA | 1752  1819 |

**Table S5:** Primers for validation of genomic modifications in *F. avenaceum*. T1 primers were combined with the RF-3 primer and T2 with PKS12-A3/A4-T1.

| **Gene** | **Primer** (5’ to 3’) | **Product (bp)** |
| --- | --- | --- |
| FaPKS5-TF1–T1 | TTGTTGGTCCGTAGAAGCATTGA | 806 bp |
| FaPKS5-TF1–T2 | TACCGGAATGTTCCTATATT | 540 bp |
| FaPKS7-TF1-T1 | TGGAAGCTGCACAATTGAGACA | 805 bp |
| FaPKS7-TF1-T2 | CCCTATTTCGGGACGATCTAGAACA | 502 bp |
| FaPKS8-TF1-T1 | ACTCAAGTACCTCGTCGGACAACT | 828 bp |
| FaPKS8-TF1-T2 | TGTACGATGCCAAATGAGGTGTTAA | 513 bp |
| FaPKS8-TF2-T1 | CTTCTAAGCCAGGCTTCTGTCG | 882 bp |
| FaPKS8-TF2-T2 | TTCAAAGGTGTCGGTCTGGTGAT | 513 bp |
| FaPKS8-TF3-T1 | TCGGGAAAGTCGGCGATAA | 858 bp |
| FaPKS8-TF3-T2 | CTTAACTTCTAAAGGGGATT | 502 bp |
| FaPKS11-TF1-T1 | AAAGCTCTGGATGCAACCGAC | 868 bp |
| FaPKS11-TF1-T2 | TTCCGGCTAACCCATTGTAACG | 502 bp |
| FaPKS11-TF2-T1 | TGAGTTGCTTCTCTACGGTTTGC | 874 bp |
| FaPKS11-TF2-T2 | CATGTGGGTTAAGTTTGGATTGGAG | 500 bp |
| FaPKS11-TF3-T1 | ACGCAGCAAACTCATTAATCAATG | 908 bp |
| FaPKS11-TF3-T2 | ACGCGACAAACTACGGAGTAGACAT | 517 bp |
| FaPKS15-TF1-T1 | AGCATTCGTATGGCTGGAAGG | 855 bp |
| FaPKS15-TF1-T2 | CGGTGCTGCAATTATGCTGACA | 527 bp |
| FaPKS26-TF1-T1 | CCGACGTATCTGCAATATAGTCAGAAAT | 923 bp |
| FaPKS26-TF1-T2 | TTAGTCTCAGCGGCAACCATCA | 537 bp |
| FaPKS27-TF1-T1 | ACTCAGCTTCCTGCACTGTGAGA | 906 bp |
| FaPKS27-TF1-T2 | AAGTCCATTTTCATCCATCGCG | 500 bp |
| FaPKS27-TF2-T1 | CGTTACCTAGACAAGACCCCGCTT | 868 bp |
| FaPKS27-TF2-T2 | TGACGGTTGGGTTTCTAAAGGGA | 504 bp |
| FaPKS27-TF3-T1 | ACAATTGGCGGCGAGGCGTA | 853 bp |
| FaPKS27-TF3-T2 | CCTCACTTCATTTCTATCCTGCCAG | 504 bp |
| FaPKS38-TF1-T1 | TCTTGTTTTTGCACAGCCTGC | 863 bp |
| FaPKS38-TF1-T2 | AAAAACATCCAGTGCACACACCC | 505 bp |
| FaPKS40-TF1-T1 | ATATGCATCCTTTGTGTTGGACAA | 858 bp |
| FaPKS40-TF1-T2 | ATCGACTTGCTCTAACGTCTTGCC | 500 bp |
| FaPKS40-TF2-T1 | CGGATGCTCAACCAGATTGCAG | 855 bp |
| FaPKS40-TF2-T2 | TGCCCTGAAATTGCCGAACA | 535 bp |
| FaPKS41-TF1-T1 | GATAATAGCCTAGGATCGCTTTCATC | 869 bp |
| FaPKS41-TF1-T2 | AGATCGCGAGCCTCGTCTATGG | 509 bp |
| FaPKS42-TF1-T1 | GCCACGTACTTGTATAAAGCAATTGAATAACAC | 1102 bp |
| FaPKS42-TF1-T2 | CAAAGCTGACTGTGCTCACAAATGT | 520 bp |
| FaPKS42-TF2-T1 | ATCGGAGCCAACGGGTGGATCA | 861 bp |
| FaPKS42-TF2-T2 | TCCGTAGCGTTGATGTTGCACA | 509 bp |
| FaPKS42-TF3-T1 | GCACTTCAATCATACTAAATTCCCAAGAAGCTT | 1087 bp |
| FaPKS42-TF3-T2 | GTCCTTCATATCGGCGAGGTATACA | 506 bp |
| FaPKS43-TF1-T1 | CAGTCCCATTTACATACAAGTCTCGC | 859 bp |
| FaPKS43-TF1-T2 | GAACCAGGTTTCCACGATAATACCA | 515 bp |
| FaPKS44-TF1-T1 | CGCTCAAGTCTTTCGTCACCAT | 812 bp |
| FaPKS44-TF1-T2 | CCCTTTGTTTTTTAGCTTGTTGCAG | 505 bp |
| FaPKS44-TF2-T1 | TTCTGGGGTACAGGGTCTCGTGCAG | 868 bp |
| FaPKS44-TF2-T2 | TGACCCTAACAGAGCCAGGTGAG | 505 bp |
| FaPKS46-TF1-T1 | AACGGTGACGGCCCCATATA | 840 bp |
| FaPKS46-TF1-T2 | GCCACTCAGACATTGTCCTTTCAA | 510 bp |
| FaPKS45-TF1-T1 | ATTGTAGCAGAGGCCCCGTTGACC | 862 bp |
| FaPKS45-TF1-T2 | ATTTCCCACCCCAATTACATCTTGT | 500 bp |
| FaPKS49-TF1-T1 | GGAGTCATAGAGAAAACACCGTACCT | 825 bp |
| FaPKS49-TF1-T2 | ATATCGGAATGCTTTCGAGCCC | 529 bp |
| FaPKS47-TF1-T1 | CCTTGGGAATCTCAATCTGCCAT | 856 bp |
| FaPKS47-TF1-T2 | CAAATGCTAATTCCTGTTAGGTGGG | 523 bp |
| FaPKS47-TF2-T1 | GGGATCGGGTCAATTCCTTGT | 854 bp |
| FaPKS47-TF2-T2 | CACCTTTCTCAGATACCCAGCAATG | 515 bp |
| FaPKS48-TF1-T1 | GGACATGTATCCATCAGTGGGGA | 863 bp |
| FaPKS48-TF1-T2 | GATGTTTGCTGACACCTGTAGTGCA | 506 bp |
| FaPKS48-TF2-T1 | GTAGCTGGGAGTTTGAATTGATCTG | 859 bp |
| FaPKS48-TF2-T2 | TAAACACGCTTGCTACGGCATG | 524 bp |
| PKS12-A3/A4-T1 | AAACTTTGCCGACATTCAGGAACT | Combine with T2 |

**Table S6:** Vector construction efficiency with the USER-Brick approach. Following USER cloning 5 transformants were analysed by colony-PCR with primers targeting the gene specific part of the individual constructs. For each construct type a single transformant was selected and subjected to restriction enzyme digestion to verify that the size and restriction fragments was as expected based on the *in silico* design. All plasmids that had tested positive for the insert specific fragments in the colony PCR test also displayed the expected restriction fragment pattern.

| **Type of construct and no. of DNA fragment in USER cloning** | **Gene name** | **Accession no.** | **# of tested colonies** | **# of correct** |
| --- | --- | --- | --- | --- |
| Deletion,  5 fragments | FaPKS3 | FA03082.2 | 5 | 5 |
|  | F**g**PKS3 | FGSG_09182 | 5 | 5 |
|  | FaPKS5 | FA11674.2 | 5 | 5 |
|  | FaPKS6 | FA08709.2 | 5 | 5 |
|  | FaPKS15 | FA12691.2 | 5 | 5 |
|  | FaPKS27 | FA10284.2 | 5 | 5 |
|  | FaPKS41 | FA07658.2 | 5 | **3** |
|  | FaPKS42 | FA07835.2 | 5 | 5 |
|  | FaPKS43 | FA07921.2 | 5 | 5 |
|  | FaPKS44 | FA11699.2 | 5 | 5 |
|  | FaPKS45 | FA08391.2 | 5 | 5 |
|  | FaPKS47 | FA08496.2 | 5 | 5 |
|  | FaPKS48 | FA10226.2 | **4** | **4** |
|  | FaPKS50 | FA04649.2 | 5 | 5 |
|  | FaPKS51 | FA04674.2 | 5 | 5 |
| In locus overexpression,  6 fragments | FaPKS5 | FA11674.2 | 5 | 5 |
|  | FaPKS15 | FA12691.2 | 5 | 5 |
|  | FaPKS27 | FA10284.2 | 5 | **2** |
|  | FaPKS41 | FA07658.2 | 5 | 5 |
|  | FaPKS42 | FA07835.2 | 5 | 5 |
|  | FaPKS43 | FA07921.2 | 5 | 5 |
|  | FaPKS44 | FA11699.2 | 5 | 5 |
|  | FaPKS45 | FA08391.2 | 5 | 5 |
|  | FaPKS47 | FA08496.2 | **4** | **4** |
|  | FaPKS48 | FA10226.2 | 5 | 5 |
|  | FaPKS50 | FA04649.2 | 5 | 5 |
|  | FaPKS51 | FA04674.2 | 5 | 5 |
| Ectopic expression,  5 fragments  (continues on next page)  (continued)  Ectopic expression,  5 fragments | FaPKS5-TF1 | FA11665.2 | 5 | 5 |
|  | FaPKS7-TF1 | FA07185.2 | 5 | 5 |
|  | FaPKS8-TF1 | FA02817.2 | 5 | 5 |
|  | FaPKS8-TF2 | FA02822.2 | 5 | **3** |
|  | FaPKS8-TF3 | FA02831.2 | 5 | 5 |
|  | FaPK11-TF1 | FA04705.2 | 5 | 5 |
|  | FaPKS11-TF2 | FA04710.2 | 5 | 5 |
|  | FaPKS11-TF3 | FA04714.2 | 5 | 5 |
|  | FaPKS15-TF1 | FA12686.2 | 5 | 5 |
|  | FaPKS26-TF1 | FA02207.2 | 5 | 5 |
|  | FaPKS27-TF1 | FA10277.2 | 5 | 5 |
|  | FaPKS27-TF2 | FA10291.2B | 5 | 5 |
|  | FaPKS27-TF3 | FA10292.2 | 5 | **4** |
|  | FaPKS41-TF1 | FA07646.2 | 5 | 5 |
|  | FaPKS42-TF1 | FA07842.2 | 5 | 5 |
|  | FaPKS42-TF2 | FA07844.2 | 5 | 5 |
|  | FaPKS42-TF3 | FA07848.2 | 5 | 5 |
|  | FaPKS43-TF1 | FA07914.2 | 5 | 5 |
|  | FaPKS44-TF1 | FA11693.2 | 5 | 5 |
|  | FaPKS44-TF2 | FA11694.2 | 5 | 5 |
|  | FaPKS46-TF1 | FA11712.2 | 5 | 5 |
|  | FaPKS45-TF1 | FA08397.2 | 5 | 5 |
|  | FaPKS49-TF1 | FA08722.2 | 5 | 5 |
|  | FaPKS47-TF1 | FA08504.2 | 5 | 5 |
|  | FaPKS47-TF2 | FA08507.2 | 5 | 5 |
|  | FaPKS48-TF1 | FA10220.2 | 5 | 5 |
|  | FaPKS48-TF2 | FA10221.2 | 5 | 5 |
|  | FaPKS51-TF1 | FA04676.2 | 5 | 5 |
|  | FaPKS52-TF1 | FA07763.2 | 5 | 5 |
|  | FaPKS52-TF2 | FA07780.2 | 5 | 5 |
|  | | | | |
| **Type** | **Total tested** | **Total correct** | **Average** | |
| Deletion, 5 fragments | 74 | 72 | 97% | |
| *In locus* overexpression, 6 fragments | 59 | 56 | 94,9% | |
| Ectopic expression, 5 fragments | 150 | 174 | 98% | |
| **Total** | 416 | 403 | 96.9% +/-0.32 | |

**Table S7:** Results from PCR based genotyping of the constructed *F. avenaceum* PKS deletion and *in locus* overexpression strains. The genotyping strategy and placement of the used primer pairs are described in details in the materials and method section of the main text. Strains carrying deletions were analysed using four primer pairs (marker, target locus and the two borders of insert) and *in locus* overexpression strains with three (marker and the two borders of the insert). Class 1 = ectopic integration; Class 2 = single crossover event; Class 3 = targeted integration (double crossover)

| **Deletions** | **Gene name** | **Accession no.** | **# of tested colonies** | **Genotype class** | | | **# of correct** |
| --- | --- | --- | --- | --- | --- | --- | --- |
|  |  |  |  | **1** | **2** | **3** |  |
|  | FaPKS5 | FA11674.2 | 9 | 1 | 1 | 7 | 7 |
|  | FaPKS6 | FA08709.2 | 10 | 2 | 1 | 7 | 7 |
|  | FaPKS15 | FA12691.2 | 12 | 1 | 3 | 8 | 8 |
|  | FaPKS27 | FA10284.2 | 10 | 1 | 2 | 7 | 7 |
|  | FaPKS41 | FA07658.2 | 10 | 0 | 1 | 9 | 9 |
|  | FaPKS42 | FA07835.2 | 10 | 1 | 2 | 7 | 7 |
|  | FaPKS43 | FA07921.2 | 10 | 0 | 2 | 8 | 8 |
|  | FaPKS44 | FA11699.2 | 10 | 1 | 1 | 8 | 8 |
|  | FaPKS45 | FA08391.2 | 10 | 2 | 1 | 7 | 7 |
|  | FaPKS47 | FA08496.2 | 10 | 0 | 1 | 9 | 9 |
|  | FaPKS48 | FA10226.2 | 8 | 1 | 1 | 6 | 6 |
|  | FaPKS50 | FA04649.2 | 10 | 2 | 5 | 3 | 3 |
|  | FaPKS51 | FA04674.2 | 10 | 1 | 2 | 7 | 7 |
|  | Total: | | 129 | 13 | 23 | 93 | 93 |
|  | In % | |  | 10.1% | 17.8% | 72.1% | **72.1%** |
|  | | | | | | | |
| ***In locus* overexpression** | **Gene name** | **Accession no.** | **# of tested colonies** | **Genotype class** | | | **# of correct** |
|  |  |  |  | **1** | **2** | **3** |  |
|  | FaPKS5 | FA11674.2 | 10 | 3 | 0 | 7 | 7 |
|  | FaPKS15 | FA12691.2 | 10 | 1 | 0 | 9 | 9 |
|  | FaPKS27 | FA10284.2 | 10 | 2 | 0 | 8 | 8 |
|  | FaPKS41 | FA07658.2 | 10 | 1 | 0 | 9 | 9 |
|  | FaPKS42 | FA07835.2 | 10 | 2 | 0 | 8 | 8 |
|  | FaPKS43 | FA07921.2 | 10 | 1 | 0 | 9 | 9 |
|  | FaPKS44 | FA11699.2 | 8 | 2 | 0 | 6 | 6 |
|  | FaPKS45 | FA08391.2 | 9 | 1 | 0 | 8 | 8 |
|  | FaPKS47 | FA08496.2 | 10 | 2 | 0 | 8 | 8 |
|  | FaPKS48 | FA10226.2 | 10 | 2 | 0 | 8 | 8 |
|  | FaPKS50 | FA04649.2 | 10 | 2 | 1 | 7 | 7 |
|  | FaPKS51 | FA04674.2 | 9 | 1 | 0 | 8 | 8 |
|  | Total: | | 116 | 20 | 1 | 95 | 95 |
|  | In % | |  | 17.2% | 0.9% | 81.9% | **81.9%** |

**Table S8:** Results from PCR based genotyping of the constructed *F. avenaceum* strains expressing PKS associated transcription factors from random loci in the genome. The genotyping was based on two primer pairs: Start = amplification of the junction between the *PgpdA* promoter and start of the TF-CDS, End = amplification of the junction between end of the TF-CDS and T-DNA backbone next to the border sequence.

| **Gene name** | **Accession no.** | **# of tested colonies** | **Genotype test** | | **# of correct** |
| --- | --- | --- | --- | --- | --- |
|  |  |  | **Start** | **End** |  |
| FaPKS5-TF1 | FA11665.2 | 3 | 2 | 2 | 2 |
| FaPKS7-TF1 | FA07185.2 | 3 | 3 | 2 | 2 |
| FaPKS8-TF1 | FA02817.2 | 3 | 3 | 3 | 3 |
| FaPKS8-TF2 | FA02822.2 | 1 | 1 | 1 | 1 |
| FaPKS8-TF3 | FA02831.2 | 3 | 3 | 3 | 3 |
| FaPK11-TF1 | FA04705.2 | 3 | 2 | 1 | 1 |
| FaPKS11-TF2 | FA04710.2 | 3 | 3 | 3 | 3 |
| FaPKS11-TF3 | FA04714.2 | 2 | 2 | 2 | 2 |
| FaPKS15-TF1 | FA12686.2 | 2 | 2 | 2 | 2 |
| FaPKS26-TF1 | FA02207.2 | 2 | 2 | 2 | 2 |
| FaPKS27-TF1 | FA10277.2 | 3 | 3 | 2 | 2 |
| FaPKS27-TF2 | FA10291.2B | 2 | 2 | 2 | 2 |
| FaPKS27-TF3 | FA10292.2 | 3 | 3 | 3 | 3 |
| FaPKS41-TF1 | FA07646.2 | 1 | 1 | 1 | 1 |
| FaPKS42-TF1 | FA07842.2 | 2 | 2 | 2 | 2 |
| FaPKS42-TF2 | FA07844.2 | 3 | 3 | 3 | 3 |
| FaPKS42-TF3 | FA07848.2 | 3 | 3 | 2 | 2 |
| FaPKS43-TF1 | FA07914.2 | 2 | 2 | 1 | 1 |
| FaPKS44-TF1 | FA11693.2 | 2 | 2 | 2 | 2 |
| FaPKS44-TF2 | FA11694.2 | 2 | 2 | 2 | 2 |
| FaPKS46-TF1 | FA11712.2 | 3 | 2 | 2 | 2 |
| FaPKS45-TF1 | FA08397.2 | 2 | 2 | 2 | 2 |
| FaPKS49-TF1 | FA08722.2 | 2 | 2 | 2 | 2 |
| FaPKS47-TF1 | FA08504.2 | 2 | 2 | 2 | 2 |
| FaPKS47-TF2 | FA08507.2 | 3 | 3 | 2 | 2 |
| FaPKS48-TF1 | FA10220.2 | 3 | 3 | 2 | 2 |
| FaPKS48-TF2 | FA10221.2 | 3 | 3 | 3 | 3 |
| FaPKS51-TF1 | FA04676.2 | 3 | 3 | 2 | 2 |
| FaPKS52-TF1 | FA07763.2 | 3 | 3 | 3 | 3 |
| FaPKS52-TF2 | FA07780.2 | 3 | 3 | 3 | 3 |
| Total: | | 75 | 72 | 64 | 64 |
| In % | |  | 96.0% | 85.3% | 85.3% |
